# *SCL*, *LMO1* and *Notch1* Reprogram Thymocytes into Self-Renewing Cells

**DOI:** 10.1371/journal.pgen.1004768

**Published:** 2014-12-18

**Authors:** Bastien Gerby, Cedric S. Tremblay, Mathieu Tremblay, Shanti Rojas-Sutterlin, Sabine Herblot, Josée Hébert, Guy Sauvageau, Sébastien Lemieux, Eric Lécuyer, Diogo F. T. Veiga, Trang Hoang

**Affiliations:** 1 Institute of Research in Immunology and Cancer – University of Montreal, Montreal, Quebec, Canada; 2 Molecular Biology Program, Faculty of Medicine, University of Montreal, Montreal, Quebec, Canada; 3 Maisonneuve-Rosemont Hospital. Montreal, Quebec, Canada; 4 Clinical Research Institute of Montreal (IRCM), Montreal, Quebec, Canada; 5 Department of Biochemistry, Faculty of Medicine, University of Montreal, Montreal, Quebec, Canada; 6 Department of Pharmacology, Faculty of Medicine, University of Montreal, Montreal, Quebec, Canada; Cincinnati Children's Hospital Medical Center, United States of America

## Abstract

The molecular determinants that render specific populations of normal cells susceptible to oncogenic reprogramming into self-renewing cancer stem cells are poorly understood. Here, we exploit T-cell acute lymphoblastic leukemia (T-ALL) as a model to define the critical initiating events in this disease. First, thymocytes that are reprogrammed by the SCL and LMO1 oncogenic transcription factors into self-renewing pre-leukemic stem cells (pre-LSCs) remain non-malignant, as evidenced by their capacities to generate functional T cells. Second, we provide strong genetic evidence that SCL directly interacts with LMO1 to activate the transcription of a self-renewal program coordinated by LYL1. Moreover, LYL1 can substitute for SCL to reprogram thymocytes in concert with LMO1. In contrast, inhibition of E2A was not sufficient to substitute for SCL, indicating that thymocyte reprogramming requires transcription activation by SCL-LMO1. Third, only a specific subset of normal thymic cells, known as DN3 thymocytes, is susceptible to reprogramming. This is because physiological NOTCH1 signals are highest in DN3 cells compared to other thymocyte subsets. Consistent with this, overexpression of a ligand-independent hyperactive *NOTCH1* allele in all immature thymocytes is sufficient to sensitize them to SCL-LMO1, thereby increasing the pool of self-renewing cells. Surprisingly, hyperactive *NOTCH1* cannot reprogram thymocytes on its own, despite the fact that *NOTCH1* is activated by gain of function mutations in more than 55% of T-ALL cases. Rather, elevating *NOTCH1* triggers a parallel pathway involving *Hes1* and *Myc* that dramatically enhances the activity of *SCL-LMO1* We conclude that the acquisition of self-renewal and the genesis of pre-LSCs from thymocytes with a finite lifespan represent a critical first event in T-ALL. Finally, *LYL1* and *LMO1* or *LMO2* are co-expressed in most human T-ALL samples, except the cortical T subtype. We therefore anticipate that the self-renewal network described here may be relevant to a majority of human T-ALL.

## Author Summary

Deciphering the initiating events in lymphoid leukemia is important for the development of new therapeutic strategies. In this manuscript, we define oncogenic reprogramming as the process through which non-self-renewing progenitors are converted into pre-leukemic stem cells with sustained self-renewal capacities. We provide strong genetic evidence that this step is rate-limiting in leukemogenesis and requires the activation of a self-renewal program by oncogenic transcription factors, as exemplified by *SCL* and *LMO1*. Furthermore, NOTCH1 is a pathway that drives cell fate in the thymus. We demonstrate that homeostatic NOTCH1 levels that are highest in specific thymocyte subsets determine their susceptibilities to oncogenic reprogramming by *SCL* and *LMO1*. Our data provide novel insight into the acquisition of self-renewal as a critical first step in lymphoid cell transformation, requiring the synergistic interaction of oncogenic transcription factors with a cellular context controlled by high physiological NOTCH1.

## Introduction

An important attribute of stem cell populations is the capacity to self-renew indefinitely both in normal development and during the process of cell transformation. Cancer stem cells, initially identified in acute myeloblastic leukemias [1], can self-renew indefinitely to propagate and maintain the disease [2]. This led to the experimental definition of leukemia initiating cell (LIC) characterized by their capacities to initiate the disease in transplanted host mice [1], [3]. Important questions remain to be resolved with regards to the nature of the cell of origin of cancer, that is the normal cells from which cancer originates [4]–[6] and the mechanisms that drive the transition to an initiated state [7]. It was initially thought that the capacity for self-renewal of LICs, also referred to as leukemic stem cells (LSCs), is conferred by the cell of origin of cancer, that is, primitive hematopoietic stem/progenitor cells (HSPCs), even though the leukemic phenotype is manifest in differentiating myeloblasts [3]. Alternatively, oncogenes acting on committed progenitors can induce a stem cell gene signature [8], leading to the reprogramming of non-self-renewing progenitors into pre-leukemic stem cells (pre-LSCs) [9], [10]. Nonetheless, only subsets of progenitors are susceptible to oncogenic reprogramming, raising questions on the molecular events that determine the susceptibility of target cells to oncogenes.

Normal thymic progenitors have limited if any self-renewal capacity [11], [12]. Bone marrow-derived progenitors settle in the thymus and gradually acquire T cell characteristics while losing “stemness” [13]. The NOTCH1 pathway is a master regulator of thymopoiesis acting at several steps, in particular at the DN3 stage where NOTCH1 together with the pre-TCR drives irreversible T-lineage commitment [14]. *NOTCH1* gain-of-function mutations were found in more than half of human T-ALL [15] and in most mouse models [16], [17]. The significance of *Notch1* for oncogenic transformation has been well established whereas the role of *Notch1* in hematopoietic stem cell (HSC) self-renewal has been controversial (reviewed in [18]). NOTCH activity is highly context-dependent [19]. Hence, a hyperactive *Notch1* allele (*NICD*; hereafter referred to as the *Notch1* oncogene) is shown to cause an exhaustion of HSCs at the expanse of T-LSCs [20]. Once transformed, LICs in *Notch1*-induced T-ALL depend on continued Notch1 signals for maintenance [15], [21]–[23] and on several downstream effectors that include *Hes1* [24]–[28] and Myc [27], [29]. These LICs were found in the immature single positive (ISP8) population, raising the question whether or not ISP8 are the cell of origin of T-ALL. Moreover, Notch1 is a weak tumor initiator [30]. Finally, the importance of *Notch1* in pre-LSCs remains to be clarified.

Self-renewal in normal HSCs is controlled by a network of transcription factors [31]. This network includes the basic helix-loop-helix (bHLH) transcription factors SCL/TAL-1 [32], [33] and the highly homologous LYL1 [34]. Both SCL [35] and LYL1 form DNA binding heterodimers with E-proteins (e.g. E2A and HEB) that are also bHLH factors and directly interact with nuclear co-factors LIM-only (LMO) proteins to form transcription complexes that drive lineage-specific gene expression in hematopoietic cells [36], [37]. SCL is partly redundant with LYL-1 in HSCs [34]. *SCL*, *LYL1* and *LMO1/2* expression decreases drastically at early stages of T-cell differentiation [13]. Their ectopic expression in the thymus, commonly driven by chromosomal rearrangements, is associated with T-ALL [38].

Overexpression of *LMO1* or *LMO2* in the thymus induces leukemia in mice with low penetrance and long latency [39]. This results from the emergence of pre-LSCs with altered gene expression [9]. Strikingly, T-ALL onset is accelerated by genetic collaboration with SCL [40], [41]. How SCL induces T-ALL remains to be clarified. Indeed, two mechanisms have been proposed for *SCL*-mediated leukemogenesis. SCL heterodimerizes with and inhibits the activity of E-proteins [42]–[44] [45]–[47], in particular of E2A and HEB that are nodal regulators in the T lineage (reviewed in [48], [49]). Accordingly, SCL inhibitory activity is sufficient to cause differentiation arrest in both B- [50] and T lineages [51]. Inhibition of E protein and differentiation blockade were, however, insufficient for leukemogenesis since most SCL transgenic lines did not develop T-ALL [40], [51], [52], with the exception of one transgenic model [53], [54]. In parallel, inhibitor of DNA-binding ID1 that sequesters E2A/HEB away from DNA was found to induce T-ALL in transgenic mice [55]. This led to the current view that bHLH oncogenic transcription factors that include SCL (or TAL1), TAL2 and LYL1 form inactive transcriptional complexes that induce T-ALL via inhibition of E proteins (reviewed in [49], [56]). With the predicament that cancer development is a Darwinian evolutionary process, the natural selection for genetic variants in which E proteins are inhibited should involve a variety of mechanisms, upregulation of bHLH transcription factors, of ID1-4 proteins that sequester E proteins away from DNA and/or inactivation of E protein encoding genes. The absence of the two latter categories so far in human T-ALL samples argues in favor of the second hypothesis, that transcription activation by oncogenic bHLH factors is an important leukemogenic driver. In support of this hypothesis, there is evidence for target gene activation in leukemic T cells [9], [57]–[60]. Nonetheless, how the SCL-LMO1/2 collaboration establishes a pre-leukemic state to initiate T-ALL remains ill-defined. Recently, *Lyl1* gene invalidation is shown to abrogate *LMO2* self-renewal activity in pre-LSCs, suggesting that *Lyl1* is an important downstream target of *LMO2* [61]. However, overexpressing *LYL1* on its own is clearly insufficient for thymocyte reprogramming [9], indicating that the molecular context for cell transformation and/or thymocyte reprogramming by *LYL1* remains to be uncovered. The inability of *SCL* or *LYL1* to induce T-ALL on their own and the long latency required for *LMO1/2-*induced leukemogenesis strongly support the view that oncogene cooperativity drives synergistic modulation of gene expression, associated with major change in cellular reorganization [62]. Understanding the process of oncogene cooperativity in leukemia initiation can reveal mechanisms that control the growth of leukemic stem cells [63].

Recent genome-wide studies of leukemic samples at diagnosis have been highly informative on the mutational process and potential driver mutations in acute leukemias [64], [65]. These powerful approaches did not allow for a clear distinction between initiating events in leukemogenesis and collaborating events that contribute to disease progression, which were revealed through two distinct approaches, the study of rare monochorionic twins [10] or of mouse models. Major questions remain nonetheless to be investigated. For example, it is not clear what determines the nature of the target cells of oncogenic reprogramming [5].

We used converging genome-wide approaches together with molecular and genetic approaches to provide novel evidence how the necessary collaboration between *SCL, LMO1* and *Notch1* determines the target cells of transformation in T-ALL and to identify novel mechanisms by which these oncogenes cooperate to activate stem cell genes and to convert normal thymocytes into self-renewing pre-LSCs. In particular, transcription activation posits a requirement for direct SCL-LMO1 interaction to assemble transcription activation complexes at target loci. In the present study, we generated transgenic mice expressing a mutant SCL that is unable to associate with LMO1/2 but retains its capacity to inhibit E2A/HEB, to provide genetic evidence for the importance of transcription activation in thymocyte reprogramming and in leukemogenesis.

## Results

### *SCL* and *LMO1* oncogenes confer an aberrant self-renewal to DN3 pre-leukemic thymocytes

**Figure 1:**
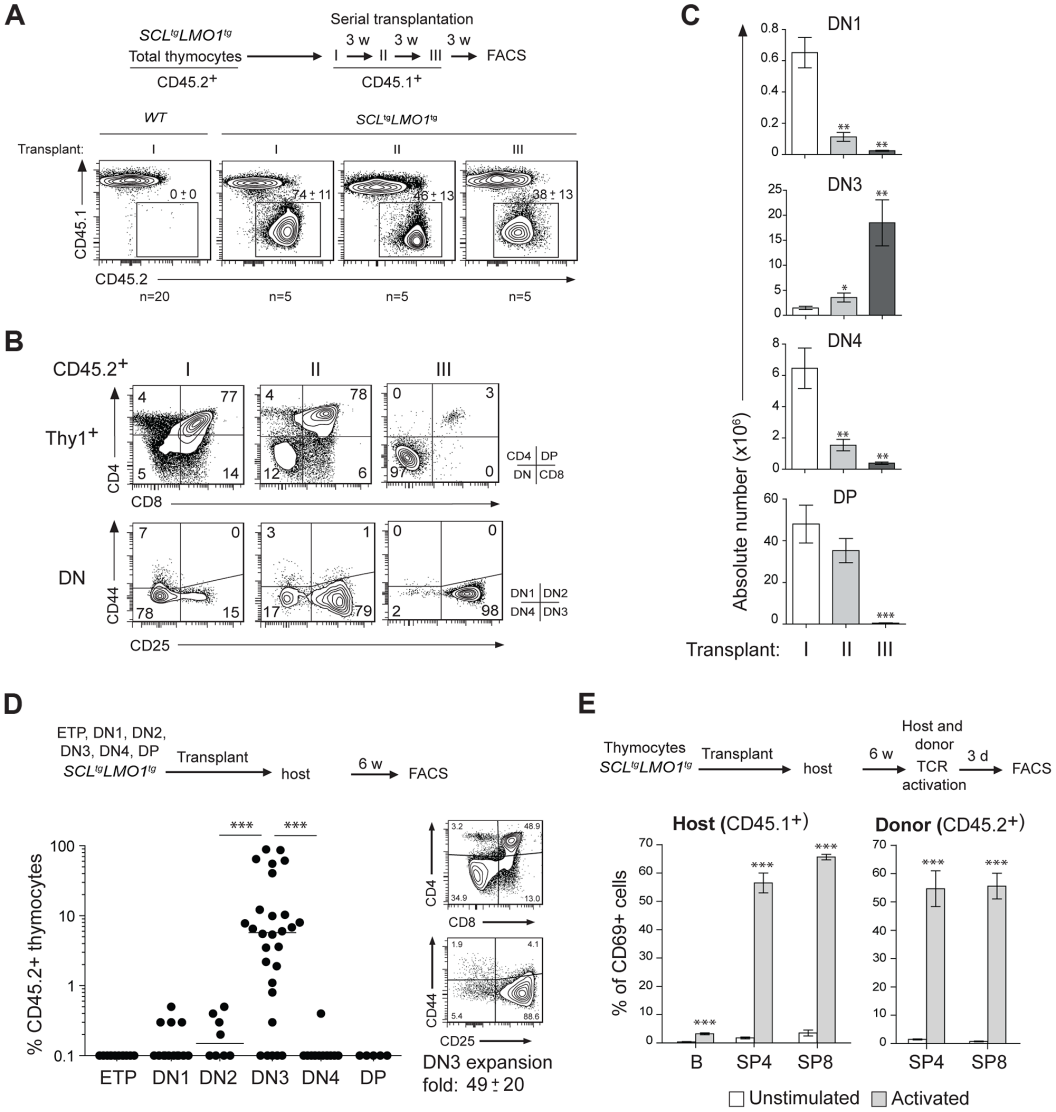
The *SCL* and *LMO1* oncogenes confer an aberrant self-renewal potential to DN3 pre-leukemic thymocytes. (**A–C**) Pre-leukemic *SCL* ^tg^ *LMO1* ^tg^ thymocytes exhibit an aberrant self-renewal potential. Pre-leukemic *SCL* ^tg^ *LMO1* ^tg^ thymocytes (CD45.2^+^) were serially transplanted into primary (I), secondary (II) and tertiary (III) recipient mice (CD45.1^+^) (1.5×10^7^ cells/mouse, 5 mice per group). Donor-derived thymocytes (CD45.1^-^CD45.2^+^) in the thymus were analyzed by flow cytometry 3 weeks after each transplantation. Note the absence of engrafment of wild type (WT) thymocytes when transplanted into primary mice (A). Immunophenotype of donor-derived thymocytes was assessed by flow cytometry (FACS) (B) and the absolute numbers of donor-derived DN1, DN3, DN4 and DP thymocytes were calculated after each transplantation (C). (**D**) *SCL-LMO1*-induced self-renewal activity is almost exclusively present in DN3 thymocytes. Purified thymocyte subpopulations (ETP, DN1-4, DP) from *SCL* ^tg^ *LMO1* ^tg^ mice were transplanted (5×10^4^ cells per mouse). Recipient mice were analyzed for engraftment as above (*left panel*). Representative flow cytometry profiles of thymocytes generated by transplanted DN3 cells (right panel). There was a net 49-fold amplification of DN3 cells in vivo. (**E**) Engrafted *SCL* ^tg^ *LMO1* ^tg^ thymocytes generate functional T cells in vivo that respond to TCR activation. Purified T cells were stimulated (activated) or not (control) with anti-CD3/CD28 beads and analyzed within the donor-derived SP4 and SP8 cells for expression of the CD69 activation marker. Host T and B cells served as positive and negative controls, respectively.

The capacity for sustained self-renewal is best observed in serial transplantation assays. While normal thymocytes did not engraft in transplanted hosts, *SCL* ^tg^ *LMO1* ^tg^ thymocytes afforded thymic reconstitution which was sustained through three serial transplantations (**Fig. 1A–C** **)**. Thymocyte differentiation in the thymus progresses from the double negative stages (DN1-4) to the CD4^+^CD8^+^ double positive (DP) stage and finally mature single positive CD4^+^ (SP4) or CD8^+^ (SP8) cells (**S1A,B Fig.**). In primary and secondary transplantation, donor-derived cells retained a capacity to give rise to DP cells. However, after the tertiary transplantation, the proportion of donor-derived DN3 thymocytes increased markedly (**Fig. 1B**), resulting in a cumulative 75-fold amplification (**Fig. 1C**). In contrast, the other thymocyte subsets decreased during the same time-frame. We transplanted purified ETP, DN1-4 and DP populations from pre-leukemic *SCL* ^tg^ *LMO1* ^tg^ mice (**Fig. 1D**). Only purified DN3 cells efficiently engrafted the thymus of recipient mice, (*left panel*). A fraction of mice transplanted with DN1 and DN2 cells exhibited less than 1% engrafment and were “negative” by definition, although this was different from the absence of engrafment from DP cells. Furthermore, purified DN3 thymocytes retained the capacity to differentiate *in vivo* into DP and SP cells and, at the same time, to expand and self-renew (*right panel*).

Interestingly, donor-derived SP4 or SP8 thymocytes recovered from transplanted mice were activated by TCR stimulation to the same extent as normal host thymocytes by upregulating the CD69 activation marker (**Fig. 1E**). This indicates that engrafted *SCL* ^tg^ *LMO1* ^tg^ thymocytes were non-leukemic. Accordingly, transplanted mice remained aleukemic, with small thymi and normal spleen size, despite the elevated expansion of DN3 thymocytes (**S1C Fig.**). Together, our results indicate that the *SCL* and *LMO1* oncogenes reprogram DN3 thymocytes into pre-LSCs that have acquired de novo self-renewing activity and retained their capacity to differentiate into functional T cells.

### The activity of SCL-LMO1 in DN3 thymocytes is sensitive to NOTCH levels

**Figure 2:**
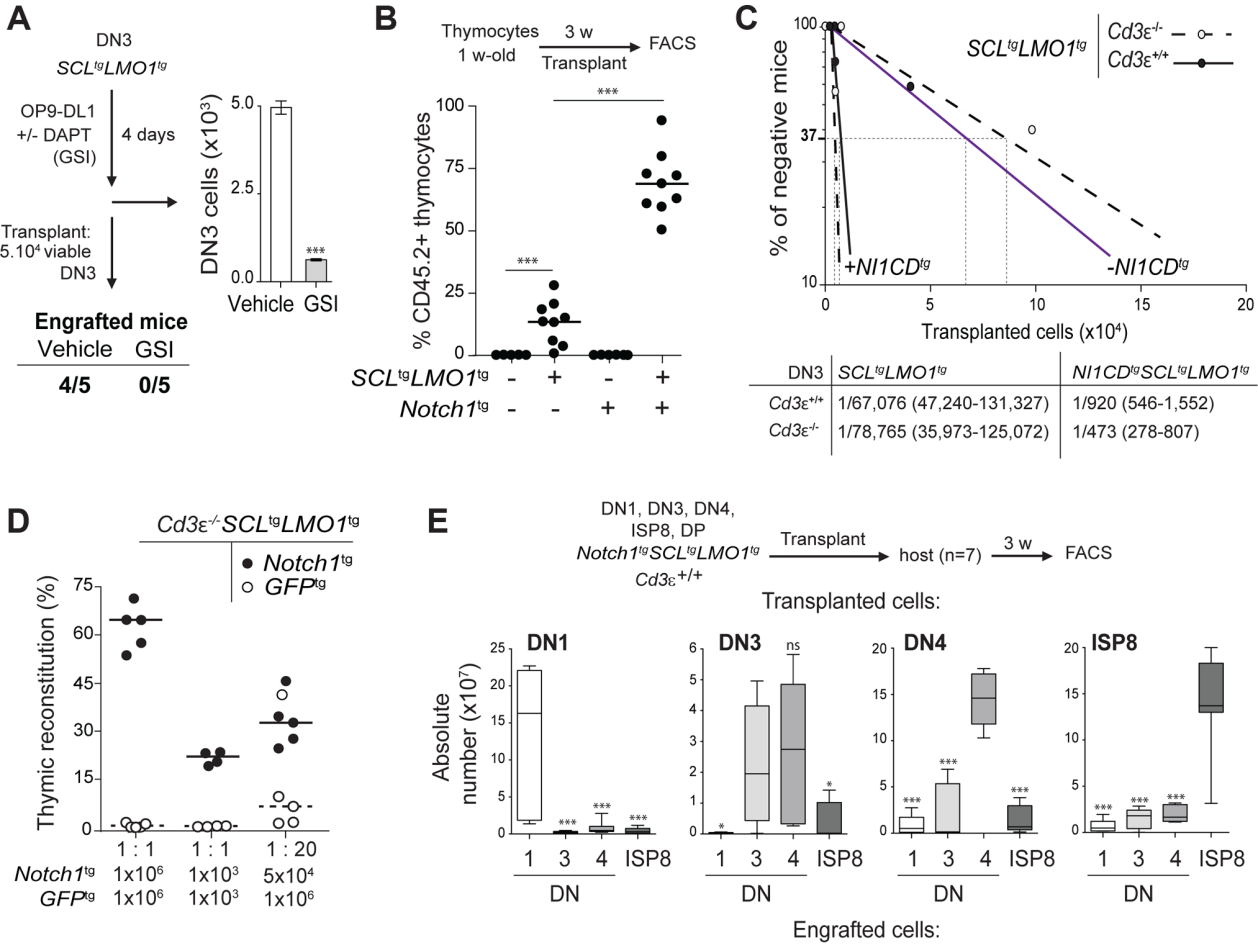
Notch1 collaborates with SCL-LMO1 to increase the pool of pre-LSCs and their competitiveness independently of a functional pre-TCR. (**A**) The engraftment of *SCL-LMO1* DN3 thymocytes is abrogated by γ-secretase inhibitor (GSI) treatment prior to transplantation. DN3 thymocytes were purified from pre-leukemic *SCL* ^tg^ *LMO1* ^tg^ mice and co-cultured on OP9-DL1 stromal cells in the presence or absence (vehicle) of 2.5 µM DAPT (GSI) for 4 days. The total numbers of viable cells recovered per culture are shown (*right panel*). Following drug treatment, equal numbers of viable cells were transplanted (5×10^4^ per mouse, n = 5). Engrafted mice: number of positive mice showing thymic reconstitution per group. (**B**) A hyperactive *Notch1* allele is insufficient to induce aberrant self-renewal in thymocytes but significantly enhances the engraftment of *SCL* ^tg^ *LMO1* ^tg^ thymocytes. Total thymocytes (1.5×10^7^) from 1-week-old mice of the indicated genotype were transplanted; recipient mice were analyzed for thymic engraftment 3 weeks later. (**C**) Oncogenic *Notch1* increases the frequencies of *SCL-LMO1* pre-LSCs independently of a functional pre-TCR. Purified DN3 thymocytes from *SCL* ^tg^ *LMO1* ^tg^ and *Notch1* ^tg^ *SCL* ^tg^ *LMO1* ^tg^ mice with (*Cd3ε* ^+/+^) or without (*Cd3ε* ^-/-^) a functional pre-TCR were transplanted in limiting dilution assays (*upper panel*). Mice were scored positive when T-cell lineage reconstitution was more than 1%; pre-LSC frequencies and confidence intervals (*lower panel*) were calculated by applying Poisson statistics using the Limiting Dilution Analysis software (StemCell Technologies). (**D**) *Cd3ε* ^-/-^ *Notch1* ^tg^ *SCL* ^tg^ *LMO1* ^tg^ pre-leukemic thymocytes outcompete *Cd3ε* ^-/-^ *SCL* ^tg^ *LMO1* ^tg^ thymocytes. Reconstitution by *Cd3ε* ^-/-^ *Notch1* ^tg^ *SCL* ^tg^ *LMO1* ^tg^ (CD45.2^+^ GFP^-^, closed circles) and *GFP* ^tg^ *Cd3ε* ^-/-^ *SCL* ^tg^ *LMO1* ^tg^ (CD45.2^+^ GFP^+^, open circles) thymocytes transplanted with the indicated cell numbers at 1∶1 or 1∶20 ratio. (**E**) *Notch1* expands the cellular targets of *SCL-LMO1* to DN1-4 and ISP8 cells. Pre-leukemic thymocyte subsets (DN1-4, ISP8 and DP) were purified from *Notch1^tg^SCL^tg^LMO1* ^tg^ mice and transplanted at 5×10^4^ cells per recipient mouse. The absolute numbers of donor-derived DN1-4 and ISP8 cells was calculated for each transplantation.

The DN3 stage in the thymus marks T-lineage commitment driven by NOTCH1 acting in concert with the pre-TCR. We therefore addressed the question whether these two pathways contribute to DN3 cell reprogramming by *SCL-LMO1.* We first addressed the functional importance of NOTCH1 in this process by lowering or increasing NOTCH activity. The expansion of pre-leukemic *SCL-LMO1* DN3 cells was recapitulated in vitro by co-culture on OP9 stromal cells expressing the NOTCH ligand Delta-like-1 (OP9-DL1) [66] (**Fig. 2A**). DAPT, an inhibitor of the -secretase, abrogated this expansion (**Fig. 2A**) without affecting the viability of the OP9-DL1 stromal cells (**S2 Fig.**). Strikingly, DAPT-treated DN3 cells were no longer able to engraft compared to control cells exposed to the vehicle alone when transplanted at equal numbers, suggesting that physiologic Notch1 signaling is required for SCL-LMO1 activity. We then addressed the consequences of supraphysiologic *Notch1* signaling on thymocyte reprogramming. Oncogenic *Notch1* has well established functions in leukemia induction and leukemia maintenance (reviewed in [18]). Nonetheless, the role of *Notch1* during this initial transition stage from a cell with finite life span to an aberrantly self-renewing pre-LSC remains to be addressed. Surprisingly, pre-leukemic *Notch1* ^tg^ thymocytes did not repopulate the thymus of recipient mice (**Fig. 2B**). Rather, *Notch1* significantly enhanced the engraftment of *SCL* ^tg^ *LMO1* ^tg^ thymocytes (**Fig. 2B**). These cells also became independent of the thymic microenvironment (**S3A–B Fig.**). Therefore, *Notch1* acts as a strong enhancer of SCL-LMO1 self-renewal activity but lacks intrinsic reprogramming activity in the absence of other oncogeneic transcription factors.

### *Notch1* oncogene confers a proliferative advantage to *SCL-LMO1*-induced pre-LSCs independently of a functional pre-TCR

To determine whether the *Notch1* oncogene modifies the frequency of *SCL-LMO1* pre-LSCs and/or their expansion at the clonal level, we performed limiting dilution assays using DN3 pre-leukemic thymocytes (**Fig. 2C**). A hyperactive *Notch1* allele increased by 60-fold the frequency of *SCL-LMO1-*induced pre-LSCs (**Fig. 2C**). In contrast, *Notch1* did not significantly modify the expansion potential of individual pre-LSC when transplanted at ∼1 competitive repopulating unit, (**S3C Fig.**). Therefore, *Notch1* expands the pool of *SCL* ^tg^ *LMO1* ^tg^ pre-LSCs *in vivo*. We took advantage of the *Tcrβ* gene rearrangement as a clonal mark to assess the diversity of pre-LSCs in transplantation assays (**S3D Fig.**). Pre-leukemic thymocytes were polyclonal before transplantation. Engrafted *SCL* ^tg^ *LMO1* ^tg^ thymocytes exhibited an oligoclonal signature whereas *Notch1* ^tg^ *SCL* ^tg^ *LMO1* ^tg^ thymocytes remained polyclonal after transplantation. Furthermore, we ruled out the possibility that *SCL* ^tg^ *LMO1* ^tg^ thymocytes had acquired *Notch1* mutations (**S1 Table**). These results indicated that a limited number of *SCL-LMO1* expressing clones were able to self-renew in the absence of *Notch1* while multiple clones were able to self-renew in the presence of *Notch1*.

We next addressed the role of the pre-TCR in the self-renewal activity induced by *SCL-LMO1* and *Notch1*. We exploited the *Cd3ε* ^-/-^ genetic mouse model in which thymocyte differentiation is blocked at the DN3a stage because of a non-functional pre-TCR/TCR (**S1 Fig. and S4A Fig.**). We observed that pre-TCR signalling did not modify the frequency of *SCL-LMO1*-induced pre-LSCs nor the genetic collaboration between *Notch1* and *SCL-LMO1* in thymocyte reprogramming (**Fig. 2C**). Moreover, the transplantation of pre-leukemic *Cd3*ε^-/-^ *SCL* ^tg^ *LMO1* ^tg^ thymocytes resulted in thymic reconstitution in primary, secondary and tertiary recipient mice (**S4B Fig.**, *left panel*), associated with DN3 cell expansion over serial transplantations (**S4B Fig.**, *right panel*), as observed with *SCL* ^tg^ *LMO1* ^tg^ thymocytes (**Fig. 1C**).

We therefore took advantage of *Cd3ε* ^-/-^ mice to specifically assess the effects of the *Notch1* transgene. We transplanted *SCL* ^tg^ *LMO1* ^tg^ thymocytes in competition with *Notch1* ^tg^ *SCL* ^tg^ *LMO1* ^tg^ thymocytes. The formers were marked with GFP to distinguish between the two cell types. Strikingly, the hyperactive *Notch1* allele conferred a marked competitive advantage to *SCL* ^tg^ *LMO1* ^tg^ pre-leukemic thymocytes when transplanted at equal concentrations both at the limiting (1×10^3^) and higher (1×10^6^) cell doses (**Fig. 2D and S** **4C Fig.**). *SCL* ^tg^ *LMO1* ^tg^ *Gfp* ^tg^ thymocytes became competitive only when transplanted at 20-fold excess. These results indicate that oncogenic *Notch1* confers a competitive advantage to *SCL* ^tg^ *LMO1* ^tg^ pre-LSCs.

### Constitutive *Notch1* activation expands the pool of *SCL-LMO1*-induced pre-LSCs to all DN-ISP8 populations

In addition, the capacity of *Notch1* ^tg^ *SCL* ^tg^ *LMO1* ^tg^ thymocytes to engraft was no longer confined to DN3 but was found in all DN subsets (DN1-DN4) and immature single-positive CD8 (ISP8) cells but not in DP thymocytes (**S4D Fig.**). Strikingly, these purified DN-ISP8 thymocytes preferentially gave rise to the same populations in transplantation, indicative of self-renewal activity (**Fig. 2E**). Therefore, elevating *Notch1* activity was sufficient to convert all immature thymocytes (DN1 to ISP8) into cellular targets of *SCL-LMO1* reprogramming activity. This expansion of cellular targets concur with the limiting dilution assay indicating that *Notch1* increased the frequency of pre-LSCs.

We conclude that NOTCH1 levels determine the expressivity of *SCL-LMO1* in thymocyte reprogramming.

### The *Notch1-Hes1*/*Myc* pathway as an enhancer of *SCL/LMO1* self-renewal activity

**Figure 3:**
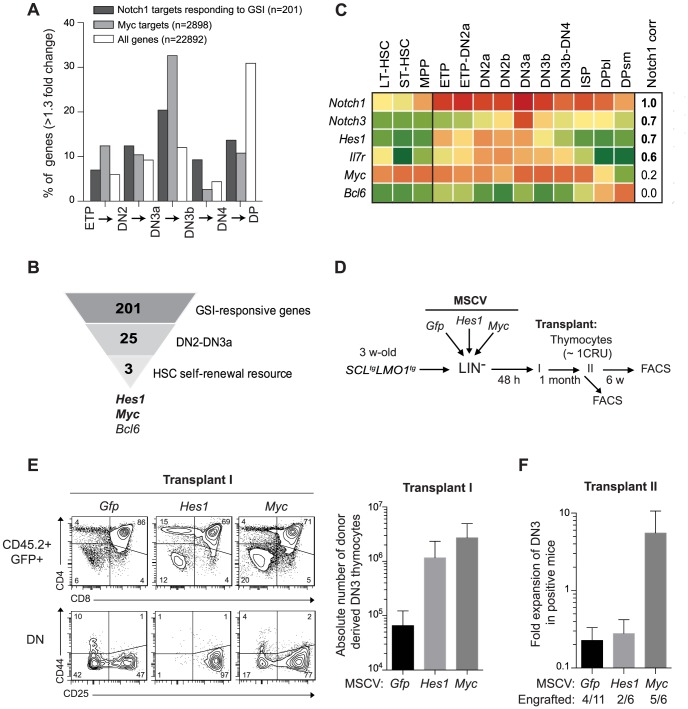
Functional importance of *Hes1* and *c-Myc* downstream of *Notch1* in thymocyte reprogramming induced by SCL-LMO1. (**A**) Expression of GSI-responsive NOTCH1 target genes during thymocyte differentiation. Global gene expression data of thymocyte subpopulations were obtained from the Immunological Genome Project (http://www.immgen.org/). The percentage of GSI-responsive NOTCH1 target genes [68] or MYC target genes [120] that are up-regulated at each transitional stage during thymocyte differentiation (>1.3-fold change) are shown. (**B**) Filtering of GSI-responsive NOTCH1 target genes that increase at the DN2 to DN3a transition and are present in HSC self-renewal resources (www.bioinfo.iric.ca/self-renewal/Main and www.bonemarrowhsc.com). (**C**) Gene expression profiles of *Notch1* and their target genes (*Notch3*, *Hes1*, *IL7r*, *Myc* and *Bcl6*) during thymocyte differentiation were collected from the Immunological Genome Project and represented as a heat map. (**D**) Schematic strategy to study the role of *Hes1* and *c-Myc* in self-renewal activity induced by *SCL-LMO1*. Lineage negative (LIN^-^) cells from *SCL* ^tg^ *LMO1* ^tg^ mice (CD45.2^+^) were transduced with either MSCV-*Hes1* and MSCV- *Myc* retroviral vectors or with control MSCV-GFP. Equal number (5×10^4^ cells) of purified GFP^+^LIN^-^ cells were then transplanted in primary mice (CD45.2^-^). Donor-derived GFP^+^CD45.2^+^ thymocytes were transplanted at the limiting dose of ∼1 CRU (10^5^ cells) per mouse into secondary recipients. (**E**) Immunophenotype of donor-derived GFP^+^CD45.2^+^ thymocytes in primary mice was analyzed by FACS (*left panel*) and the absolute number of DN3 cells was calculated (*right panel*). (**F**) The fold expansion of donor-derived GFP^+^CD45.2^+^ DN3 thymocytes was calculated in secondary mice.

Our findings indicate that SCL-LMO1 self-renewal activity is confined to the DN3 stage (**Fig. 1D**), is GSI-responsive and is sensitive to NOTCH1 levels (**Fig. 2A–B**). Interestingly, DN3 thymocytes are normally more sensitive to decreased *Notch1* gene dosage compared to earlier thymocyte progenitors [67]. We therefore capitalized on the comprehensive gene expression data from the Immunological genome project (Immgen) together with NOTCH1 ChIP-Seq data [68] and HSC self-renewal resources to inform about candidate genes in pre-LSC self-renewal. First, we investigated the upregulation pattern of NOTCH1-bound genes that are GSI-responsive during early thymocyte differentiation. Considering genes that increased by more than 1.3-fold at each transitional stage, the analysis revealed that the percentage of up-regulated NOTCH1-bound genes steadily increased from the ETP to the DN3a stage and decreased thereafter (**Fig. 3A** **and S2 Table**) as expected (reviewed in [69]). The general trend was also observed for the total transcriptome but the magnitude of the effect was stronger for the NOTCH1-bound genes (**Fig. 3A**). Furthermore, NOTCH1-bound genes sharply decreased at the DP stage when the total transcriptome increased. Finally, DN3 cells in WT and *SCL-LMO1* mice exhibit the highest levels of *Notch1* and *Notch3* genes and of the NOTCH reporter activity in Transgenic Notch Reporter (*TNR* ^tg^) mice (**S5A–B Fig.**) as reported [23]. Therefore, NOTCH activity was highest in DN3 thymocytes, coinciding with the self-renewal activity of *SCL-LMO1*. *MYC* has been implicated downstream of *NOTCH1* in T-ALL [27]. Interestingly, we found that the increase in MYC target genes coincided with that of NOTCH1 and peaked at the DN2-DN3a transition (**Fig. 3A**).

Candidate genes operating with SCL-LMO1 at the DN3 stage should also be GSI-responsive, as engraftment by SCL-LMO1 DN3 thymocytes was DAPT-sensitive. These genes should operate prior to pre-TCR signalling, i.e. at the DN3a stage, since *SCL-LMO1*-induced self-renewal activity was fully efficient in *Cd3ε*-deficient DN3 thymocytes (**Fig. 2C and S** **4B Fig.)**. Based on the list of GSI-responsive NOTCH1-bound genes published by Wang et al [68], 25 were found to increase at the DN2 to DN3a transition (**S2 Table**). We next intersected this short list with HSC self-renewal resources [70], [71] and found 3 genes *Hes1, Myc* and *Bcl6* (**Fig. 3B**). We ruled out *Bcl6* because of high expression in DP cells that are resistant to cellular reprogramming while both *Myc* and *Hes1* decreased at this stage (**Fig. 3C**). We noticed that Notch1 target genes correlate well with Notch1 mRNA levels during thymocyte differentiation, except *Myc*. Despite this, the increase in MYC-bound genes at the DN2-DN3a transition correlates with that of NOTCH1-bound genes (Fig. 3A). These observations suggest MYC activity is subject to additional levels of regulation. *Myc* is a well known target of NOTCH1 in T-ALL [27], [72]. Moreover, *Hes1* overexpression expanded HSCs in culture [70], [73] whereas *Hes1* invalidation decreased LSCs in *Notch1-*induced T-ALL [24]. We therefore determined whether *Hes1* or *Myc* may be important for this new activity of *Notch1* at enhancing *SCL-LMO1* reprogramming activity.

To determine whether *Hes1* or *Myc* can substitute for *Notch1* as an enhancer of *SCL-LMO1*, we overexpressed these genes in HSCs from SCL-LMO1 transgenic mice using the MSCV retroviral vector (**Fig. 3D**). Both *Hes1* and *Myc* caused an expansion of the DN3 population in transplanted mice, which was twenty to forty fold higher than that observed with the control vector (GFP) (**Fig. 3E**). Furthermore, all DN populations were expanded by *Myc* whereas the activity of *Hes1* was more specific to the DN3 population (**Fig. 3E and S** **6A Fig.**). Thymocytes overexpressing *Hes1* or *Myc* were recovered and transplanted into secondary mice at the limiting dose of ∼1 CRU per mouse. Consistent with this limiting dose, the proportion of engrafted mice remained at 30% in the *Gfp* and *Hes1* groups (**Fig. 3F**), suggesting that the frequency of pre-LSC was not modified by *Hes1*. Nonetheless, the total number of DN3 thymocytes recovered from these mice were modestly higher with *Hes1*. In contrast, *Myc* overexpression expanded the population of DN3 and 5 of 6 mice were reconstituted, indicative of increased pre-LSC frequency (**Fig. 3F**). Therefore, *Myc* expanded DN3 thymocytes and increased their self-renewal activities, thus recapitulating the activity of the *Notch1* transgene. In comparison, *Hes1* activity was mostly in DN3 expansion. Accordingly, thymocytes from *Notch1* ^tg^ *SCL* ^tg^ *LMO1* ^tg^ mice in which *Hes1* levels were decreased by a *Hes1*-directed shRNA (**S6B Fig.**, *upper panel*) exhibited two-fold decreased regenerative capacities compared to control cells expressing the empty vector (**S6B Fig.**, *lower panel*). Moreover, the self-renewing DN and ISP8 populations were similarly decreased while DP cells that lacked self-renewal activity were unaffected (**S6B Fig.**, *lower panel*). Therefore, *Hes1* is required downstream of *Notch1* as an expansion factor, whereas *Myc* controls both self-renewal activity and cell expansion.

In summary, our results indicate that *Notch1* signal controls both *Hes1* and *Myc* and determines the capacity of DN3 thymocytes to be reprogrammed by *SCL-LMO1*.

### *SCL-LMO1* upregulated a stem cell gene signature in DN3 thymocytes

**Figure 4:**
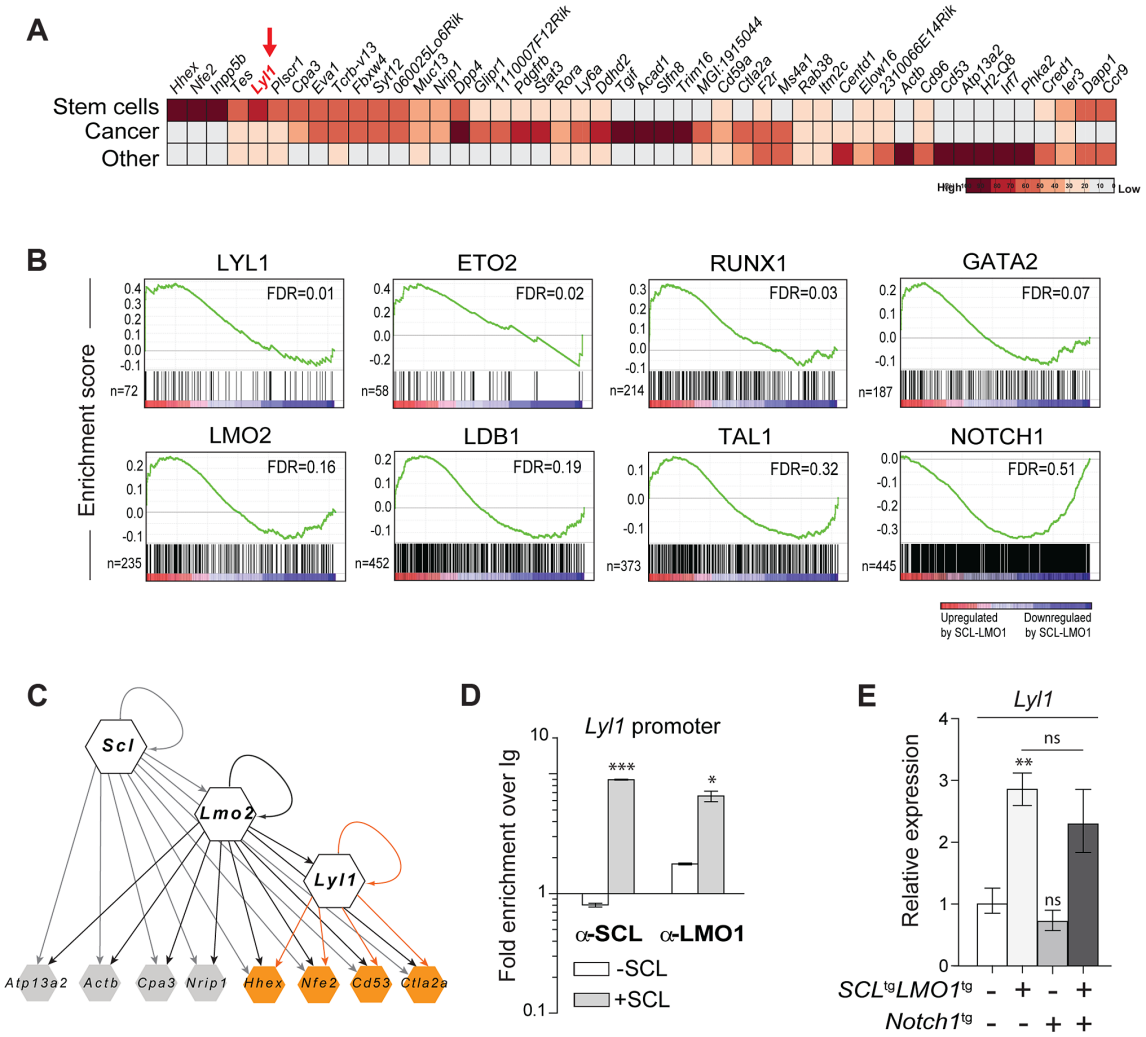
*Lyl1* coordinates a self-renewal network downstream of *SCL-LMO1*. (**A**) Analysis of SCL-LMO1-upregulated genes in *Cd3ε* ^-/-^ thymocytes. Gene signatures were analysed using the Stem Cell Discovery Engine tool as described in Experimental procedures, and signatures deemed enriched in SCL-LMO1 up-regulated genes (adjusted p-val <0.05) were classified into broad categories. The heatmap depicts the frequency of association to each gene by signature categories (stem cells, cancer, and other). (**B**) GSEA analysis of hematopoietic transcription factor signatures in *SCL-LMO1* thymocytes. The lists of genes bound by 31 hematopoietic transcription factors within 2 kb of their proximal promoters were extracted from a compendium of ChIP-seq experiments (see Materials and Methods). The top 7 transcription factors are illustrated (FDR, false discovery rate, ranging from 0.01–0.32). In comparison, NOTCH1-bound genes were not up- or down-regulated by *SCL-LMO1*. (**C**) Hierarchical organisation of the self-renewal network controlled by SCL-LMO1. Integration of published ChIP-seq data [74] with up-regulated genes in DN3 pre-leukemic thymocytes identified common targets of SCL, LMO2 and LYL1 (highlighted in yellow). Incoming edges represent the binding of regulators at the proximal promoters of target genes (peaks within 2kb of the transcription initiation sites). (**D**) SCL and LMO1 occupy *Lyl1* regulatory sequences. Chromatin extracts from the AD10.1 DN cell line expressing SCL (+SCL) or not (-SCL) were immunoprecipitated with the indicated antibodies. *Lyl1* regulatory sequences were amplified by q-PCR. Data are expressed as fold enrichment over IgG controls. (**E**) *Lyl1* gene expression is induced by *SCL-LMO1* but is not modified by *Notch1* in DN3 thymocytes. mRNA levels in purified DN3 thymocytes from the indicated transgenic mice were determined by qRT-PCR and normalized to *β-Actin* (Mean +/- SD, n = 3).

To identify candidate genes that confer self-renewal capability to pre-leukemic DN3 thymocytes, we made use of the *Cd3ε-*deficient mouse model in which oncogene-induced self-renewal activity was unaltered (**Fig. 4A**). We compared gene expression profiles of thymocytes from *SCL-LMO1* transgenic and age-matched non transgenic *Cd3ε^-/-^* mice, taken three weeks after birth. At this time point, the transcriptome analysis identified only 53 up-regulated and 33 down-regulated genes in *SCL-LMO1* expressing thymocytes (**S3 Table**), indicating that the gene expression programs in the two cell types were comparable. We compared this list of differentially expressed genes with the genome binding profiles of SCL and LMO2 in several hematopoietic cell lines identified from a compendium of ChIP-seq datasets [74]. Within the down-regulated genes, only three had SCL peaks (*Cdc6*, *Cdkn1a* and *Slc4a1*) and none are presumed LMO2 target. In contrast, 9 of the up-regulated genes are presumed direct SCL and LMO2 targets (**S7A Fig. and S3 Table**). These observations concur with the view that SCL together with LMO2 preferentially enhances transcription. We overlapped the *SCL-LMO1* up-regulated gene set with a compendium of molecular signatures (http://discovery.hsci.harvard.edu/). We found a subset of genes that are frequent in stem cell and cancer signatures (**S7B Fig. and S4 Table**), that includes *Hhex*, *Nfe2* and *Lyl1*. In particular, *Lyl1* is associated with HSC and cancer cell signature (**S7B Fig.**) and controls HSC survival [34] (**S7B Fig.**; www.bonemarrowhsc.com).

Next, we applied gene set enrichment analysis (GSEA) to uncover transcription factor signatures enriched in *SCL-LMO1* thymocytes, using a compendium of 55 ChIP-seq datasets representing 31 hematopoietic transcription factors from the HemoChIP project and others (see Materials and Methods). Surprisingly, the LYL1 signature was the most up-regulated in *SCL-LMO1*-expressing DN3 thymocytes (**Fig. 4B**). Significantly, GSEA analysis also detected an up-regulated signature of SCL transcriptional partners, GATA2, LMO2, LDB1, ETO2 and SCL, together with LYL1 and RUNX1-bound genes [75]. On the other hand, NOTCH1 signature was not significantly enriched in this gene set, concurring with the view that *SCL-LMO1* and *Notch1* operate in parallel pathways. Furthermore, all LYL1-bound genes are comprised within the SCL-LMO1-bound gene set (**S7**A **Fig.**, right panel). Overall, our transcriptome analysis predicted a hierarchy downstream of *SCL-LMO1* in which *Lyl1* could coordinate a *Notch1*-independent self-renewal network (**Fig. 4C**).

By ChIP analysis, we found that SCL occupancy of the *Lyl1* locus in SCL-expressing DN cells (+ SCL) induced a 2- to 4-fold higher LMO1 binding to the *Lyl1* promoter compared to control cells (-SCL) (**Fig. 4D**). Finally, we observed by qRT-PCR that *Lyl1* expression was significantly up-regulated by *SCL-LMO1* (**Fig. 4E**), concurring with our microarray results. In contrast, the *Notch1* oncogene did not modify *Lyl1* expression in DN3 thymocytes expressing or not *SCL-LMO1* (**Fig. 4E**).

We conclude that SCL and LMO1 induce aberrant stem cell gene expression in DN3 thymocytes and reprogram these cells to acquire stem cell-like properties.

### Inhibition of E protein is insufficient for leukemogenesis

SCL activates or represses gene expression, depending on its protein partners (reviewed in [76]). Transcription activation critically depends on direct SCL-LMO1 or -LMO2 interaction to assemble a transcription complex on DNA [36], [37]. This interaction is dispensable for transcription inhibition of E protein targets, which is directly attributed to SCL interaction with E2A or HEB.

In particular, GSEA analysis indicated that E2A-presumed targets were not enriched within the list of differentially expressed genes (**S8A Fig.**), suggesting that inhibition of E2A activity by SCL-LMO1 in DN3 thymocytes was not a major perturbation at the molecular level. We designed the *SCLm13* that is specifically defective in LMO1/2 binding while heterodimerization with E2A/HEB was unaffected [37] (**S8B Fig.**). Compared to wild type *SCL*, *SCLm13* failed to activate the transcription of *Lyl1* in transient assays whereas inhibition of E protein activity remained intact (**S8C Fig.**). We previously identified *Ptcra* as a direct target of HEB/E2A that is inhibited by SCL [46]. We therefore stably introduced SCL and SCLm13 in the DN cell line AD10 and found that both genes inhibited the expression of *Ptcra* to the same extent, indicating that direct SCL-LMO1/2 interaction was dispensable for inhibition of E proteins (**S8D Fig.**).

**Figure 5:**
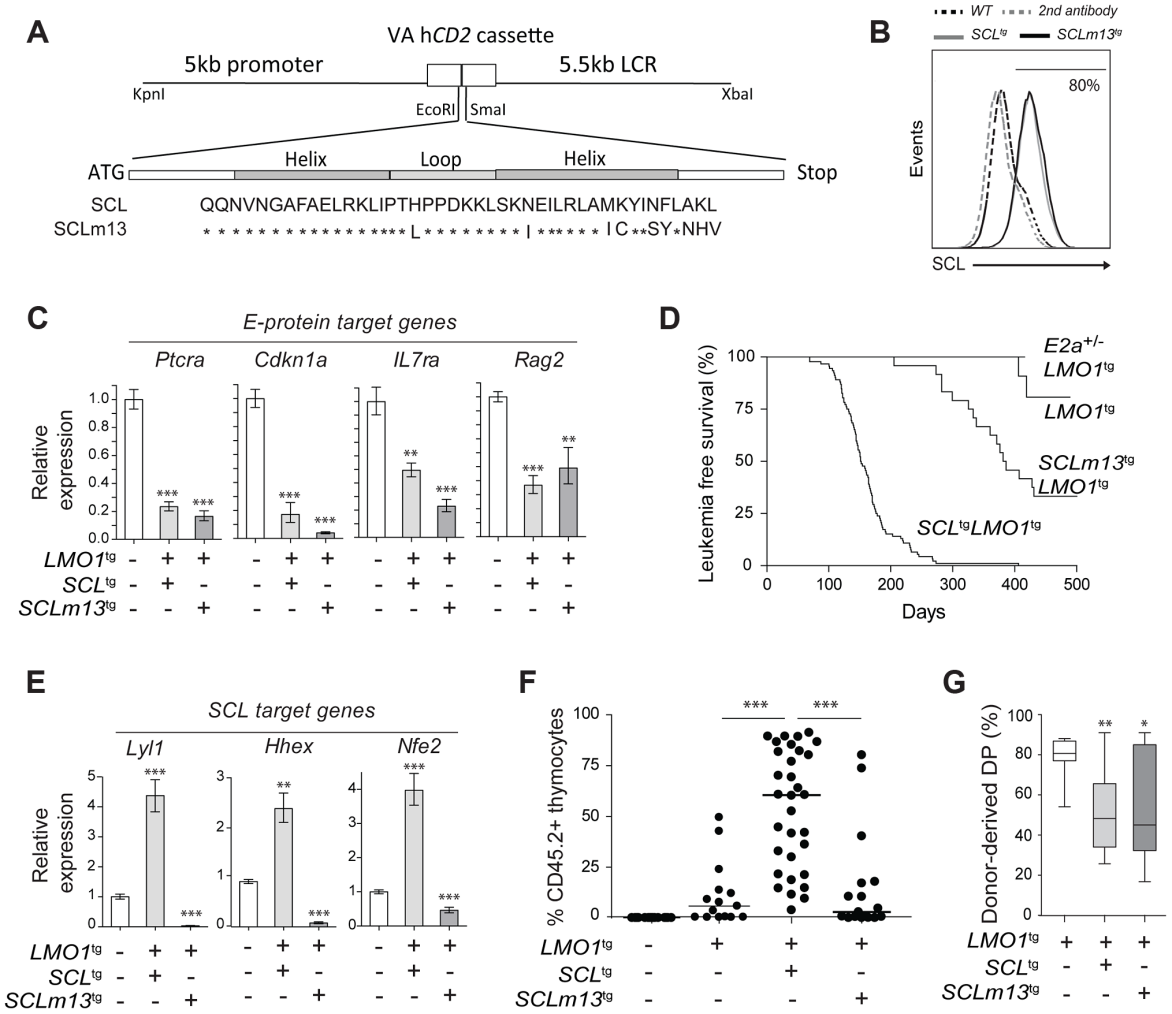
Transcription activation driven by SCL-LMO1 interaction is critical for thymocyte reprogramming and T-ALL induction. (**A**) Generation of transgenic mice expressing the LMO1-binding defective mutant SCLm13. The sequence coding for wild type human SCL or human SCLm13 HLH domain mutant [37] were cloned into the VA h*CD2* cassette to generate transgenic mice. Shown are amino acids of the HLH region of SCL or SCLm13. (**B**) Immunofluorescence of human SCL (wt or m13) by flow cytometry. Thymocytes were stained with the monoclonal antibody against human SCL (BTL73). Control cells were stained with the second antibody only. (**C**) Expression of E protein target genes is inhibited both by *SCL-LMO1* and *SCLm13-LMO1* transgenes in DN3 thymocytes. mRNA levels in purified DN3 thymocytes from the indicated transgenic mice were determined by qRT-PCR and normalized to *β-Actin* (Mean +/- SD, n = 3). (**D**) Kaplan-Meier curves of the time to leukemia for *LMO1^tg^*, *E2a^+/-^LMO1^tg^*, *SCL^tg^LMO1^tg^* and *SCLm13^tg^LMO1^tg^* mice. (**E**) The interaction between SCL and LMO1 is required to activate the transcription of the self-renewal genes *Lyl1*, *Hhex* and *Nfe2* in DN3 thymocytes. mRNA levels in purified DN3 thymocytes from the indicated transgenic mice were determined by qRT-PCR and normalized to *β-Actin* (Mean +/- SD, n = 3). (**F–G**) SCL but not the LMO1-binding defective SCL-m13 mutant collaborates with LMO1 to induce abnormal thymic reconstitution potential to thymocytes. Pre-leukemic thymocytes (1.5×10^7^ cells) from 3-week-old mice were transplanted. Recipient mice were analysed for thymic reconstitution (CD45.2^+^Thy1^+^) after 6 weeks (F) and the proportion of DP cells in engrafted CD45.2^+^Thy1^+^ thymocytes was assessed by FACS (G).

E proteins are major cell fate deteminants in the thymus [77], [78], leading to the current view that T-ALL induction by SCL-LMO1/2 is due to E protein titration and inhibition [47]. To directly address the question whether the inhibition of E2A by SCL-LMO1 was sufficient for leukemogenesis, we generated transgenic mice expressing wild type *SCL* or the *SCLm13* mutant at comparable levels (**Fig. 5A–B**). We observed that *SCLm13* fully retained its capacity to inhibit the expression of E protein target genes in DN3 thymocytes (**Fig. 5C**). Significantly, while SCLm13 still inhibited E proteins (**S8D Fig.**), there was a striking difference between the survival curves of *SCL* ^tg^ *LMO1* ^tg^ and *SCLm13* ^tg^ *LMO1* ^tg^ transgenic lines (**Fig. 5D**). *LMO1* ^tg^ mice develop T-ALL with 20% penetrance and delayed onset at 400 days, as reported [39]. In contrast, the disease was fully penetrant in *SCL* ^tg^ *LMO1* ^tg^ mice, with an accelerated onset of 170 days [17], [40]. In *SCLm13* ^tg^ *LMO1* ^tg^ mice however, leukemia onset was delayed to 380 days and the penetrance reduced to 65% (**Fig. 5D**), underscoring the importance of SCL-LMO1 interaction in leukemogenesis. To further address the question whether the genetic collaboration between SCL and LMO1 in leukemogenesis was due to inhibition of E proteins, we generated *E2a* ^+/-^ *LMO1* ^tg^ mice. Loss of one *E2a* allele significantly decreased expression levels of E2A target genes in DN thymocytes (**S8E Fig.**) but did not mirror the collaboration of the *SCL* transgene with *LMO1* to induce T-ALL. Together, our results indicate that inhibition of E2A is insufficient for leukemogenesis and that direct SCL-LMO1 interaction is an important determinant of leukemia onset and disease penetrance.

### Transcription activation by SCL-LMO1 is required for thymocyte reprogramming

We next addressed the question whether direct SCL-LMO1 interaction is required for self-renewal activity in DN3 thymocytes. The *m13* mutation severely impaired the activation of self-renewal genes including *Lyl1* (**Fig. 5E**) and drastically decreased the capacity of total thymocytes (**Fig. 5F**) or purified DN3 thymocytes (**S9A Fig.**) to reconstitute the thymus of transplanted hosts. Thymic engraftment of *SCLm13* ^tg^ *LMO1* ^tg^ thymocytes were reproducibly decreased to levels observed with *LMO1* ^tg^ only. Nonetheless, *SCLm13* retained the same capacity as *SCL* to block the DN to DP transition compared to *LMO1* alone (**Fig. 5G** **and S9B Fig.**), a transition stage controlled by *E2a* and *Heb* gene dosage [79], [80]. Together, our results indicate that inhibition of E protein and thymocyte differentiation blockade are distinct from the acquisition of self-renewal activity, which requires direct SCL-LMO1 interaction and transcription activation of a self-renewal program.

### *Lyl1* can substitute for *SCL* to collaborate with *LMO1* and reprogram thymocytes

**Figure 6:**
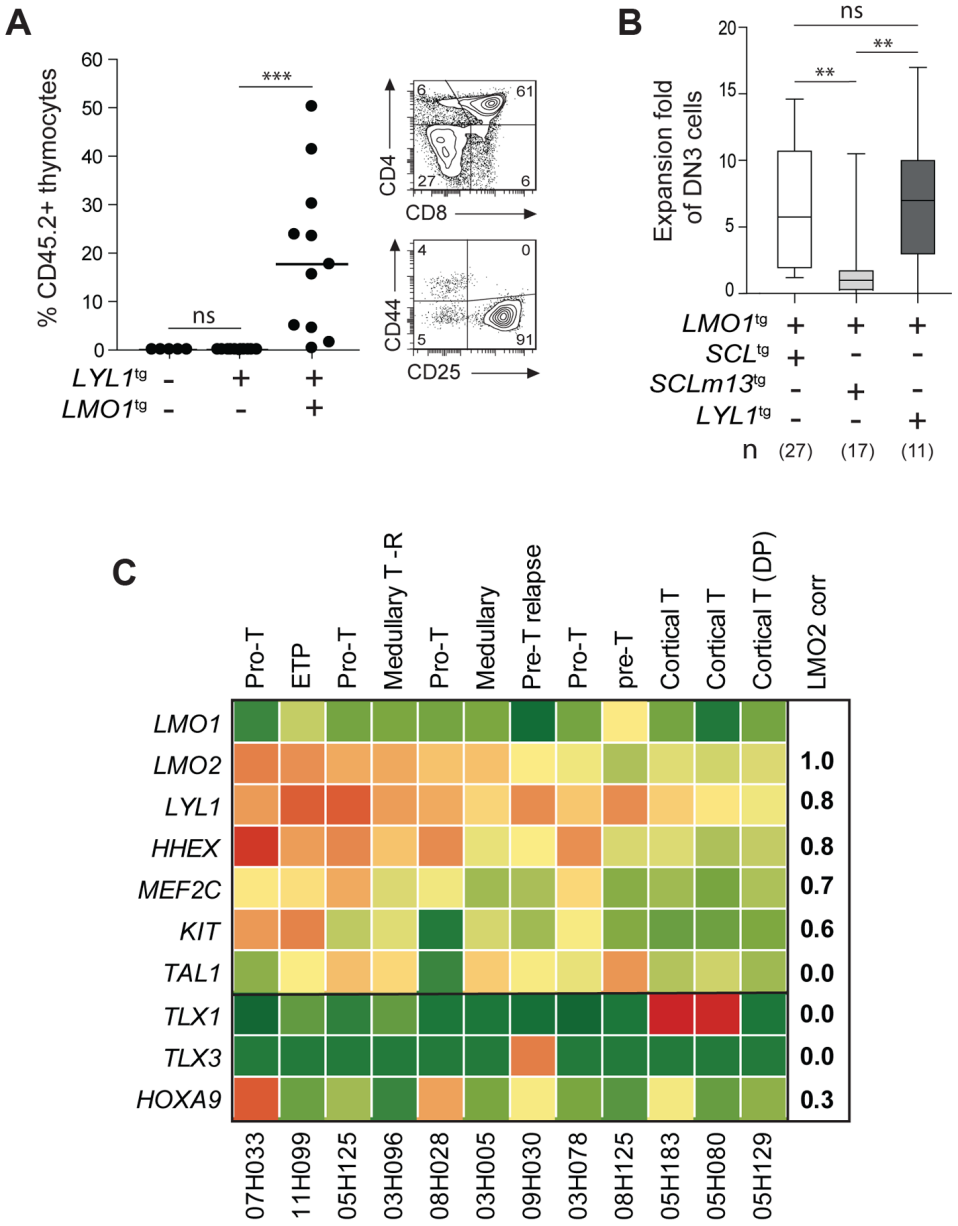
LYL1 and LMO1/2 are co-expressed in human T-ALL and collaborate to reprogram thymocytes. (**A**) *LYL1* collaborates with *LMO1* to induce abnormal thymic reconstitution potential to thymocytes. 1.5×10^7^ thymocytes from the indicated mice were transplanted and thymic engraftment was analyzed after 6 weeks (*left panel*). Representative FACS profile of engrafted *LYL1* ^tg^ *LMO1* ^tg^ thymocytes (*right panel*). (**B**) *LYL1-LMO1*-induced DN3 expansion was comparable to *SCL-LMO1*-induced expansion after transplantation, as illustrated by the box plots (with the median and extreme values of each distribution, cohorts of n mice). (**C**) LMO2 expression levels correlate with LYL1 levels in T-ALL patient samples. Illustrated are the RPKM values for the indicated human gene. Note the high correlation coefficient between *LMO2* and *LYL1* and the absence of correlation with *TLX1/3*.

Network analysis point to the importance of *Lyl1* downstream of SCL-LMO2 (**Fig. 4C**), consistent with published results [61]. Yet, ectopic expression of *Lyl1* on its own did not recapitulate *LMO2*-induced aberrant self-renewal in thymocytes [9]. We reasoned that LYL1 activity most likely requires interaction with LMO1/2 for the following reasons: (*i*) the SCL interaction interface with LMO1/2 is conserved in LYL1 [36]; (*ii*) LYL1 is in complex with SCL and LMO2 [81]; (*iii*) LYL1 binding to DNA often overlaps with SCL and LMO2 binding [75]; and (*iv*) *Lyl1* is redundant with *Scl* in controlling HSC self-renewal [34]. We therefore generated *LYL1* ^tg^ *LMO1* ^tg^ mice to address the question whether *LYL1* enhances *LMO1* self-renewal activity. *LYL1* enhanced by 3-fold the activity of *LMO1* on thymocyte engraftment (**compare** **Fig. 6A,** *left panel* and **Fig. 5F**), whereas LYL1 alone did not reprogram thymocytes as expected. Similar to *SCL-LMO1*, *LYL1-LMO1* expanded DN3 cells only after transplantation (**Fig. 6A,** *right panel and ***S** **10 Fig.**) and this expansion was in the same order of magnitude compared to the inactive *SCLm13-LMO1* (**Fig. 6B**). The virtual convergence of *SCL-LMO2* and *LYL1-LMO2* target genes (**Fig. 4C**) may explain the capacity of *LYL1-LMO1* to mimic *SCL-LMO1* in DN3 thymocytes (**Fig. 6A–B**).

By RNA-Seq of 12 T-ALL patient samples, we found that *LYL1* and *HHEX* mRNA levels are highly correlated with *LMO2* levels (r = 0.8, **Fig. 6C**), concurring with the view that *LYL1* and *HHEX* are downstream targets of *LMO2* in T-ALL.

Interestingly, *LYL1* expression in the absence of *TAL1* was found in 4 of 12 samples but *TAL1* expression was never found in the absence of *LYL1* (**Fig. 6C**). These observations concur with the view that *TAL1* is upstream of *LYL1* (**Fig. 4C–E**) and with the essential role of *Lyl1* in pre-thymic progenitors as well as in ETP-DN2 [82]. Moreover, the absence of correlation between *TAL1* and *LMO2* mRNA levels are consistent with the observations that *LYL1*, but not *TAL1*, is essential for *LMO2*-induced T-ALL [61].

We observed higher *LYL1*, *LMO2, HHEX* and *MEF2C* levels in ETP and pro-T ALL in adult (**Fig. 6C**) and pediatric (**S11 Fig.** [38]) T-ALL, consistent with this gene triad being direct targets of activation by MEF2C [83]. Nonetheless, *LYL1* and *LMO1/2* expression was detected in a majority of T-ALL samples independently of *MEF2C* or of phenotypic classification and included *TLX1/3*- and *HOXA9*-expressing leukemias (**Fig. 6C and S** **11 Fig.**). These observations suggest that the molecular pathways controlling self-renewal described here is not limited to T-ALL samples harboring *TAL1* or *LMO1/2* translocations but may be relevant to other oncogenic subtypes of T-ALL.

## Discussion

### Self-renewal as an initiating event in leukemia

**Figure 7:**
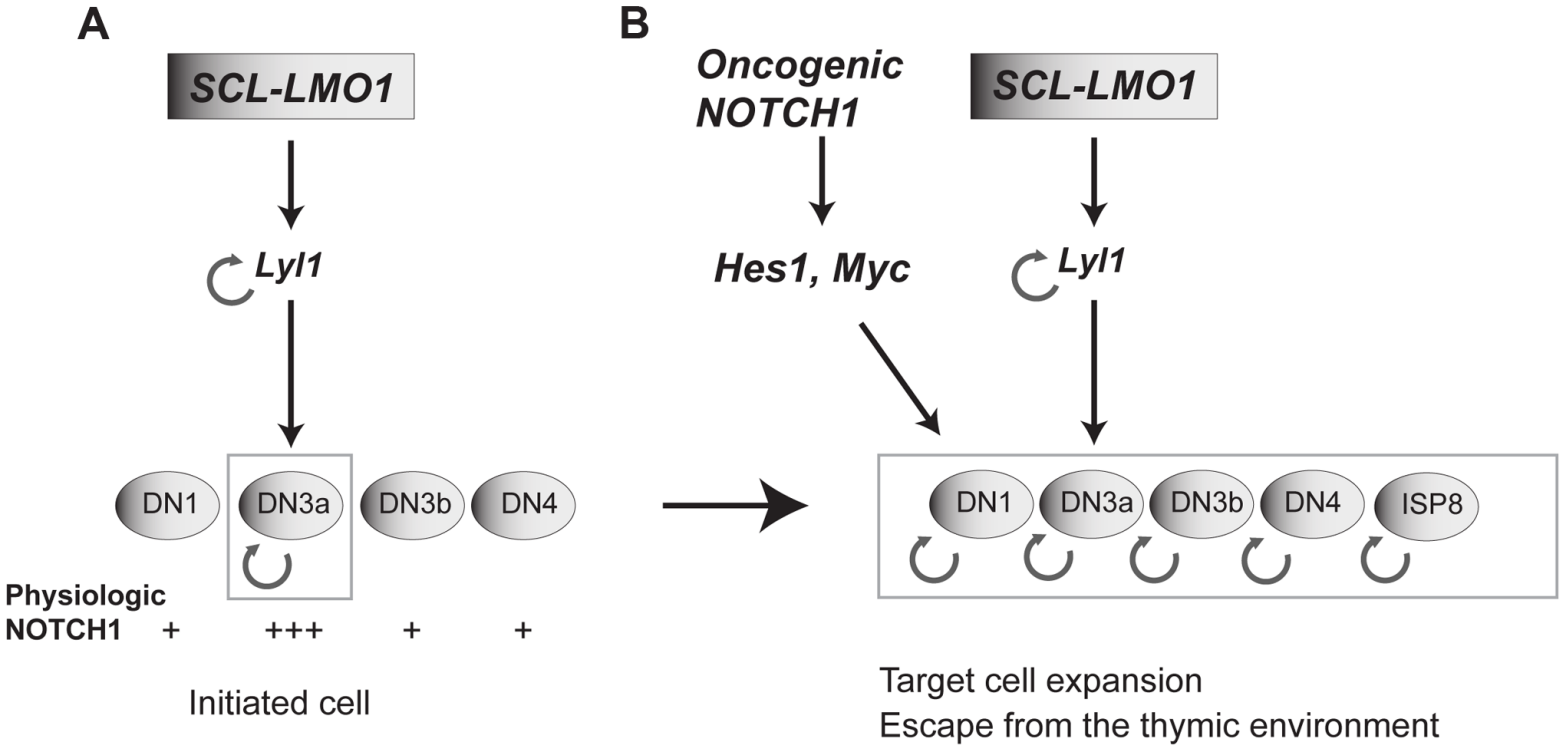
Model of the collaboration between the SCL, LMO1 and *Notch1* oncogenes. (**A**) *SCL* and *LMO1* interact to upregulate *Lyl1* gene expression and create a feed forward loop that activates self-renewal in DN3 thymocytes. DN3 cells are prone to *SCL-LMO1* self-renewal activity due to higher physiological NOTCH levels. (**B**) The *Notch1* oncogene drastically enhances *SCL-LMO1*-induced self-renewal activity to expand the pool of target cells to DN1-4 and ISP8 in a parallel pathway via *Hes1* and *c-Myc*. *SCL-LMO1* initiated cells (A) subsequently acquire gain of function *Notch1* mutations (B), causing target cell expansion and escape from thymic environmental control.

Self-renewal is a mandatory trait of cancer stem cells as drivers of clonal expansion and evolution through layers of selective pressure [7]. This self-renewal activity is essential for long-term propagation. We now provide evidence that self-renewal is an initiating event triggered by the reactivation of stem cell genes in thymocytes (**Fig. 7A**), as exemplified by chromosomal translocations driving ectopic *SCL*, *LYL1* or *LMO1/2* expression in thymocytes. Our data indicate that LYL1 coordinates a self-renewal network downstream of SCL-LMO1 to reprogram thymocytes with a finite life span into self-renewing pre-LSCs. Importantly, these self-renewal genes require the high levels of physiological NOTCH1 in DN3 thymocytes for expressivity. Their activities are therefore modulated by the thymic mircroenvironment. Furthermore, the *Notch1* oncogene is devoid of intrinsic self-renewal activity but dramatically enhances *SCL-LMO1* activity by conferring a proliferative advantage to *SCL-LMO1*-primed pre-LSCs and by recruiting all immature thymocytes into division to expand the pool of pre-LSCs (**Fig. 7B**). Consequently, the hyperactive *NOTCH1* allele acts as a strong enhancer of SCL-LMO1 by conferring additional fitness traits to *SCL-LMO*-initiated pre-LSCs, and allows for escape from envrironmental signals.

### Transcription activation by SCL-LMO1: reprogramming DN3 thymocytes into self-renewing pre-LSCs

LMO2 interaction with SCL has several consequences. First, interaction with SCL protects LMO1/2 from proteasomal degradation [37]. Second, SCL brings LMO2 to DNA, with two possible outputs: transcription activation or transcription inhibition. E proteins are major drivers of thymocyte development by activating gene expression programs that control cell survival, cell cycle and T-cell differentiation. In particular, SCL-LMO1 inhibit E protein activity and thymocyte differentiation [46], [47], leading to the current view that *SCL-LMO1/2* induced T-ALL is due to E protein inhibition [47]. We bring several lines of evidence to indicate that the inhibition of E proteins is not the major cause of T-ALL. First, within the differentially expressed gene set in SCL-LMO1 DN3 thymocytes, we found a significant enrichment for binding by all SCL transcriptional partners, whereas E2A binding was not enriched. Second, removal of one *E2a* allele did not collaborate with *LMO1* to induce T-ALL even though *E2a* was haploinsufficient for target gene expression. Third, we show that inhibition of E protein activity by the *SCLm13* mutant did not enhance *LMO1* self-renewal activity, resulting a dramatically decreased leukemogenic activity compared to wild type *SCL*, as assessed by decreased penetrance and increased latency. The modest enhancement of *LMO1* in T-ALL induction by *SCLm13* remains compatible with a tumor suppressor function for E proteins [47]. Therefore, the interaction of LMO1 with SCL, which is required to assemble a transcriptionally active complex on DNA [37], is an important determinant of T-ALL development due to the reactivation of stem cell genes in DN3a thymocytes, during the pre-leukemic stage. By network analysis of the *SCL-LMO1* transcriptome in DN3a thymocytes, we identified a hierarchy downstream of *SCL-LMO1* which is controlled by *Lyl1*. Previous work indicated that *Lyl1* is critical for the oncogenic functions of *LMO2*, consistent with a non-redundant function for *Lyl1* in lymphoid progenitors and ETP [82]. This finding not mirrored by ectopic *Lyl1* expression in the thymus [9] whereas *Hhex* deficiency [84] is mirrored by *Hhex* overexpression [9]. Considering that *LYL1* and *LMO2* chromosomal rearrangements were found simultaneously in a rare case of human T-ALL [85], we now report that *LYL1* collaborates with *LMO1* to reprogram DN3 thymocytes.

In summary, we provide genetic evidence that transcription activation by SCL and LMO1 is a major determinant of self-renewal in pre-LSCs and of the aggressiveness of T-ALL.

### *Notch1* as a strong enhancer of *SCL-LMO1* that expands the pool of pre-LSCs

NOTCH signaling is essential for T-cell commitment and specification. In particular, NOTCH1 cooperates with the pre-TCR to control cell survival and proliferation at the DN to DP transition [14], at a critical checkpoint in the thymus. We previously showed that pre-TCR activity at the DN3 stage is required for the acquisition of *Notch1* mutations in *SCL* ^tg^ *LMO1* ^tg^ thymocytes [17]. Once mutated, these hyperactive *Notch1* alleles are sufficient to drive progression to T-ALL in concert with *SCL-LMO1*. Therefore, the pre-TCR is a strong determinant of leukemia onset and of disease penetrance. Strikingly, we show here that the initiating event of reprogramming DN3 thymocytes into self-renewing pre-LSCs by SCL-LMO1 is independent of the pre-TCR but requires NOTCH1 signal. Taken together, our observations indicate that the pre-TCR is a collaborating event in disease progression but dispensable for the initial transition from DN3 cells to pre-LSCs. In contrast, we show that high levels of physiologic Notch signals in DN3 cells were required for SCL-LMO1 reprogramming activity.

Functional studies of the *NOTCH1* oncogene at time of overt leukemia in both human [21], [22] and murine LSCs [23], [86], [87] showed that NOTCH1 controls leukemia initiating cell activity. In contrast, the role NOTCH1 in HSC self-renewal was controversial [30], [88], [89]. Using the mouse model as a unique opportunity to specifically understand initiating events in T-ALL, we unexpectedly found that a hyperactive NOTCH1 allele is devoid of intrinsic reprogramming activity in thymocytes, suggesting that weaker leukemia-associated *Notch1* alleles [30] also lack this activity, similar to *Notch3* [9]. Instead, high levels of NOTCH1 activity sensitize target cells to the reprogramming activity of SCL and LMO1. Indeed, supraphysiologic NOTCH signaling was required past the DN3 stage, when physiologic NOTCH activity fell sharply. Therefore, our work provides a distinct conceptual framework to grasp the significance of the frequent co-occurrence of *NOTCH1* gain of function mutations with major classes of oncogenic transcription factors in T-ALL.

Multiple genetic interactions have been described for *Notch1* [26], [27], [90] (reviewed in [91]). Similar to *Notch1*, *Hes1* also drives T-cell development and inhibits alternate fates [92]. Interestingly, the conditional invalidation of *Hes1* in adult hematopoietic cells led to T-cell defects and disrupted T-ALL maintenance [24]. Whether *Hes1* contributes to oncogenic reprogramming of thymocytes at the initiation of the disease remained to be addressed. Here, we show that the hyperactive *Notch1* allele upregulates *Hes1* by 4-fold in DN3 thymocytes, which was insufficient for thymocyte self-renewal *in vivo* and required co-expression of the *SCL-LMO1* oncogenes.

*Myc* is required for the correct balance between self-renewal and differentiation of normal HSCs. Indeed, enforced *Myc* expression leads to HSC exhaustion whereas *Myc* deficiency results in increased HSC pool and self-renewal [93]-[95]. Our analysis of the Immgen data set indicates that MYC target genes but not *Myc* mRNA levels correlate with NOTCH1 activity during normal differentiation. This indicates additional levels of regulation; in particular MYC proteins are regulated by the ubiquitin ligase FBW7 in HSCs [96], which is frequently mutated in T-ALL patients [97]. These observations point to the critical importance of regulating MYC levels in thymocytes. *MYC* is a well-documented target of *NOTCH1* in leukemogenesis [27]. Furthermore, Myc promotes fibroblast reprogramming into induced pluripotent stem cells [98]. We now show that ectopic *My*c expression in thymocytes recapitulates the activity of the *Notch1* transgene to enhance thymocyte reprogramming by *SCL-LMO1*. Our observations on the role of *Notch1-Myc* as an enhancer of *SCL-LMO1* shed light on the pathway through which the BET-bromodomain inhibitors (JQ1) that inhibited Myc could decrease the growth of primary leukemic cells, i.e. most likely due to interference with the NOTCH1 pathway [87], [99], [100]. Finally, our observations on the primordial role of *Myc* over *Hes1* in substituting for NOTCH1 signals is consistent with the model of feed-forward-loop activated by NOTCH1 and MYC that promotes leukemic cell growth [29].

Therefore, our work clarifies the important role of *Notch1-Hes1/Myc* in the thymus as enhancers of self-renewal, but not as oncogenes with reprogramming activity. The *Scl* and *Lmo2* genes [46] are silenced in DN3 thymocytes by a repressive histone mark [13]. We therefore surmised that chromosomal translocations or retroviral integration upstream of the *LMO2* locus observed in pediatric T-ALL overcome these repressive marks to cause ectopic expression of oncogenes such as *LYL1, SCL* and *LMO2* which, in the context of DN3 thymocytes, collaborate with *NOTCH1-HES1/MYC* to confer aberrant self-renewal to these cells. We therefore propose a model in which *SCL-LMO1-Lyl1* and *Notch1-Hes1* are complementary in thymocyte reprogramming (**Fig. 7B**).

### Oncogenic reprogramming sets a pre-leukemic state by enabling self-renewal

Phenotypic plasticity or lineage infidelity is often observed in cancer [101]. A recent report indicates that phenotypic plasticity predisposes reprogrammed fibroblasts to express stem cell characteristics and to induce tumors in nude mice [102]. In contrast, we show here that pre-leukemic stem cells conserve their DN3 phenotype through three rounds of transplantation and that the acquisition of self-renewal as an essential stem cell characteristic can occur in the absence of phenotypic plasticity. Therefore, our data indicate that phenotype plasticity is not an essential premise for oncogenic reprogramming whereas self-renewal is a mandatory trait [7].

The cell of origin of T-ALL was inferred from the phenotype of the leukemic cells [38], or of LICs which was closer to the phenotype of a T-cell progenitor [22], [103]. Nonetheless, LICs. have evolved through several selective constraints and acquired additional complexity and are defined as cells that produce an overt invasive leukemia. Here we define the cell of origin of T-ALL and the mechanisms by which oncogenes reprogram normal thymocytes. We bring evidence that the activation of a self-renewal program requires collaboration between several genes and incoming environmental signals, which is likely to determine the nature of the cell of origin of leukemia. Thus, *SCL*, *LYL1* or *Notch1* are not endowed with intrinsic reprogramming activity. Both *SCL* and *LYL1* strongly enhanced *LMO1* self-renewal activity in DN3 thymocytes due to higher endogenous NOTCH1. Furthermore, the combination of three oncogenes, *Notch1*, *SCL* and *LMO1* had the strongest effect on self-renewal. Therefore, our data provide new mechanistic insights into the original two-hit model of cell transformation. Instead of each oncogene acting independently as a master switch in leukemia initiation, our work argues for the coincidence detection model in which biological outputs depend on the simultaneous occurrence of multiple signals within a network. Such cooperativity governs the process of self-renewal in pre-LSCs, which is an initiating event in T-ALL.

High LYL1 and LMO2 expression in T-ALL was previously associated with immature or ETP-ALL [38], [83], [104]. While TAL1 expression in T-ALL was linked with a late cortical stage of T cell differentiation on the basis of cell surface markers [38] or whole transcriptome [104], we provide cellular and genetic evidence that the initiating events occur in earlier stages in which NOTCH1 signals are highest, i.e. at the DN2b to DN3a transition, and that the *Cd3ε* gene is dispensable. These observations prompted us to examine the transcriptome of human adult T-ALL (Leucegene-IRIC) [105] and pediatric T-ALL [38]. This analysis also revealed that LYL1 and LMO2 were high in ETP and pro-T ALL but were detectable in almost all samples, suggesting that the molecular network defined in our study might operate in most T-ALL [106]. The importance of the SCL-LMO1 interaction described here for pre-LSC self-renewal activity, combined with the molecular view of this interaction interface suggests that targeting SCL-LMO interaction might represent a novel and promising therapeutic avenue. Such approach will be applicable to LYL1-LMO2 since the residues interacting with LMO2 are conserved between SCL and LYL1.

## Materials and Methods

### Mice and ethics statement

All animals were maintained in pathogen-free conditions according to institutional animal care and guidelines set by the Canadian Council on Animal Care. Our protocol entitled “T-cell acute lymphoblastic leukemia induced by the SCL oncogene” was approved by the Ethics Committee of experimentation on animals of the University of Montreal, CDEA (Comité de d?ontologie de l'expérimentation sur les animaux).

Transgenic mice were previously described: *pSil-TSCL* (*SCL^tg^*) [40], *Lck*-*LMO1* (*LMO1* ^tg^) and *Lck*-*NotchIC9* (*Notch1* ^tg^) (NIAID/Taconic Repository Bethesda), *E2a^+/-^* [107], *Lck*-*LYL1* (*LYL1* ^tg^) (International Mouse Strain Resource), *Transgenic Notch Reporter* (*TNR* ^tg^) (Tg(Cp-EGFP)25Gaia, The Jackson Laboratory, Maine, United States) and *Cd3ε* ^-/-^ [108]. Mice cohorts were generated by cross-breeding. Their genotypes were verified by PCR. The gene encoding the short isoform (p22) of wild-type and m13 mutant [37] SCL protein was amplified by PCR using the following primers: 5′-GCGCGAATTCATGGAGATTACTGATGGT-3′ and 5′-TATACCCGGGTCACCGAGGGCCG-GCTCC-3′. These fragments were digested with EcoRI and SmaI and subcloned in *Cd2*-VA minigene construct (gift from Dr Dimitris Kioussis, National Institute for Medical Research, London, UK) [109], [110]. DNA was microinjected into the pronucleus of C57BL6 mice by IRIC Transgenesis Core Facility, University of Montreal. Transgenic mice were backcrossed into the C57BL6 background for more than 10 generations.

### Transplantation assay

Pre-leukemic thymocytes from donor mice (CD45.2^+^) are transplanted intravenously into sub-lethally irradiated (600cGy) recipient mice (CD45.1^+^). Thymic chimerism in the T-lineage (Thy1.2^+^) was analysed by flow cytometry (FACS) and illustrated by the percentage of donor-derived cells (% CD45.2^+^) found in the recipient thymus.

### Limiting dilution assays

Pre-leukemic thymocytes from *SCL^tg^LMO1* ^tg^ and *Notch1^tg^SCL^tg^LMO1* ^tg^ mice were transplanted into sub-lethally (600 cGy) irradiated hosts (CD45.1^+^) at various cell doses (10^7^, 10^6^, 10^5^, 10^4^, 10^3^, and 10^2^) per recipient mouse (n = 7 mice for each dose). Mice were scored positive when T-cell lineage reconstitution was more than 1%. Pre-leukemic stem cell (pre-LSC) frequency (Range pre-LSC ± Confidence Interval) and Competitive Re-populating Unit (CRU) frequency for the indicated genotypes were calculated by applying Poisson statistics using the Limiting Dilution Analysis software (Stem Cell Technologies). The same strategy was used to compare the pre-LSC frequencies of DN3 *SCL* ^tg^ *LMO1* ^tg^ and *Cd3ε* ^-/-^ *SCL* ^tg^ *LMO1* ^tg^ thymocytes. The mean activity of pre-leukemic stem cells (MAS) is calculated according to the Harrison formula [111], [112]. MAS represent the pre-LSC potential of approximately 1 CRU: MAS  =  [RU]/[CRU] where RU represents the re-populating activity of pre-LSC and CRU was determined by limiting dilution analysis as above. RU was calculated as previously described [33]. Since the number of competitor cells corresponds to the number of cells in the thymus of sub-lethally irradiated recipient mice, the formula was applied as follows: RU  =  [number of donor-derived cells]/[number of competitor host cells in recipient mouse thymus].

### *In vivo* competitive assay

Pre-leukemic *Cd3ε* ^-/-^ *Notch1* ^tg^ *SCL* ^tg^ *LMO1* ^tg^ thymocytes (CD45.2^+^ GFP^-^) from one-week-old mice were mixed with *Cd3ε* ^-/-^ *Gfp* ^tg^ *SCL* ^tg^ *LMO1* ^tg^ competitor thymocytes (CD45.2^+^ GFP^+^) in two ratios (1∶1 and 1∶20) at the indicated cell doses in Fig. 2D and S4C Fig. Mixed cells were then transplanted in irradiated hosts (CD45.1^+^). Thymic reconstitution by transplanted cells was assessed by FACS analysis 3 weeks post-transplantation.

### Immunostaining and FACS analysis

Single-cell suspensions were prepared from thymi of mice of the indicated ages and genotypes. Immunostaining was done as previously described [46]. All antibodies used for flow cytometry analysis were from Pharmingen (BD Biosciences, Mississauga, Ontario, Canada): CD44 (IM7), CD25 (PC61.5), CD4 (RM4-4), CD8 (53-6.7), Thy1.2 (30-H12) and CD24 (30-F1). Dead cells were excluded by propidium iodide staining. FACS, cell cycle and cell division analysis were performed on a LSRII cytometer (BD Biosciences) using DIVA (BD Biosciences) and ModFit LT (Verity Software House, Topsham, Maine, United States) software.

### Nuclear SCL labeling

For nuclear SCL labeling, thymocytes were fixed and permeabilized with Fixation/Permeabilization Solution Kit and washed 3 times with Perm/Wash buffer (BD Cytofix/Cytoperm, 554714; BD Biosciences, Mississauga, Ontario, Canada). The cells were then labeled with the monoclonal anti-human SCL BTL73 [113] at 1∶10 dilution, washed extensively with PBS, followed by a goat anti-mouse antibody coupled to FITC. The antibody was a generous gift from Danièle Mathieu-Mahul (Institut de Génétique Moléculaire, Montpellier, France).

### Co-culture conditions

Pre-leukemic cells were purified by FACS from transgenic mice and co-cultured on (GFP-positive) OP9 and OP9-DL1 stromal cell lines, as described previously [66]. Briefly, pre-leukemic cells were co-cultured on OP9 and OP9-DL1 cells in reconstituting a-MEM medium (12561, Gibco, Life Technologies, Burlington, Ontario, Canada) supplemented with 10% FBS (12318, Gibco), HEPES 10 mM (15630-080, Gibco), sodium pyruvate 1 mM (11360-070, Gibco), b-mercaptoethanol 55 µM (21985-023, Gibco), glutamax 2 mM (15750-060, Gibco), penicillin/Streptomycin (15140-122, Gibco), 5 ng/mL FLT-3 Ligand (308-FK, R&D system) and 5 ng/mL IL-7 (407-ML, R&D system). Medium was half changed twice per week and the cells were counted and phenotyped by FACS after co-culture.

### T-cell activation assay

T-cell stimulation was assessed using anti-CD3/CD28 beads as previously described [114]. Briefly, engrafted *SCL* ^tg^ *LMO1* ^tg^ pre-leukemic T cells (donor thymocytes) and host thymocytes were purified by FACS and co-cultured on a OP9-DL1 stromal cell line over 3 days with anti-CD3/CD28 beads (Dynabeads Mouse T-Activator CD3/CD28, 114.52D, Invitrogen, Life Technologies, Burlington, Ontario, Canada). The expression of the activation marker CD69 (H1.2F3, eBioscience, San Diego, California, United States) was then analyzed by flow cytometry at the surface of SP4 and SP8 cells. Host B cells purified from the spleen were used as a negative control.

### Cell cycle assay

DN3 thymocytes from WT, *SCL* ^tg^ *LMO1* ^tg^, *Notch1* ^tg^ and *Notch1* ^tg^ *SCL* ^tg^ *LMO1* ^tg^ mice were purified by FACS and co-cultured on OP9-DL1 stromal cell line during 3 days. Derived-thymocytes were immunostained with T cell markers and then fixed and permeabilized (CytofixCytoperm Plus, BD Bioscience) during 30 minutes before the staining with the Ki67-FITC antibody. The DAPI was added at the end of the staining as a marker of DNA content. Cycle cycle analysis of DN3 thymocytes was finally analysed by FACS.

### Microarray analysis

RNAs collected from *Cd3ε* ^-/-^ and *Cd3ε* ^-/-^ *SCL* ^tg^ *LMO1* ^tg^ thymocytes were amplified and hybridized onto Affymetrix Mouse Genome 430A 2.0 arrays (Ottawa Genome Centre, Ottawa, Ontario, Canada). Raw data pre-processing and differential expression analysis was carried out using Bioconductor packages in the R environment, according to the following pipeline: (*i*) probesets were summarized and normalized using the RMA procedure implemented in the Affy package [115]; (*ii*) absent/present probesets were detected using the MAS5 implementation of the Affy package, and probesets deemed absent in both conditions (*Cd3ε^-/-^* and *Cd3ε* ^-/-^ *SCL* ^tg^ *LMO1* ^tg^) were removed from downstream analysis; and (*iii*) detection of differentially expressed genes was carried out using the Rank Products package [116].

### Collection and analysis of ChIP-Seq datasets

We collected genome-wide chromatin occupancy data for 31 hematopoietic transcription factors (51 ChIP-seq experiments in total) from Wang et al [68] and the HemoChIP project [74]. NOTCH1-binding peaks in G4A2 and T6E murine cell lines were computed using the Galaxy tool, according to the following steps: (i) sequence reads were mapped to the mouse genome mm9 using Bowtie with default parameters (maximum 2 mismatches); and (ii) peak coordinates were determined by the MACS tool, using the Pvalue cutoff <10–9. Peak coordinates for the HemoChIP dataset mapped to the mouse genome mm9 were downloaded from http://hscl.cimr.cam.ac.uk/ChIP-Seq_Compendium/ChIP-Seq_Compendium2.html. Finally, all peaks were associated to their closest transcription start sites in the mouse genome using PeakAnalyzer v.1.4 tool [117]. Gene lists bound by transcription factors used in downstream analyses (Figs. 4A, 5D, 6A) included only those genes containing at least one binding site for the given regulator within the proximal promoter (2 kb region around the transcription start site).

### RT-qPCR

Total RNAs were prepared from 50,000 purified cell population cells from 1-week-old mice using RNeasy extraction kit (Qiagen, Mississauga, Ontario, Canada). First strand cDNA syntheses were performed by reverse transcription as described [46]. Primer sequences are listed in S5 Table. Real-time quantitative PCR was done with SYBR Green Master Mix (Applied Biosystems, Foster City, California, United States) on Stratagene Mx3000 apparatus (Stratagene, La Jolla, California, United States). ΔΔCt values were calculated using Ct values from *β-actin* gene as reference.

### ChIP assays

The DN thymoma cell line AD10.1 [46] was cultured in IMDM (Invitrogen, Burlington, Ontario, Canada) containing 10% inactivated foetal calf serum (FSC) and 50 µM β-mercaptoethanol. The parental cell line was retrovirally transduced with MSCV empty vector or MSCV-SCL-expressing vector, and stable transfectants were kept under neomycin selection (1 mg/mL). Chromatin immunoprecipitation were performed as described previously [118] using the following antibodies: anti-SCL mouse monoclonal antibodies BTL73 (generously provided by Dr. D. Mathieu-Mahul, Institut de Génétique Moléculaire, Montpellier, France), rabbit anti-LMO1 (Bethyl Laboratories, A300-314A; Cedarlane Laboratories, Burlington, Ontario, Canada), and anti-rabbit IgG (Sigma, St-Louis, Missouri, United States). Oligonucleotide sequences used for promoter amplification are shown in S5 Table.

### Gene transfer into bone marrow cells

Gene transfer into bone marrow cells from 1-week-old pre-leukemic *Notch1* ^tg^ *SCL* ^tg^ *LMO1* ^tg^ mice was performed essentially as previously described [119]. Bone marrow cells were depleted of lineage positive cells through immunomagnetic bead cell separation (LIN^-^) and plated in suspension culture in IMDM with 15% FCS, 100 ng/mL murine Steel Factor (SF), 10 ng/mL human IL-6, 100 ng/ml human IL-11 and 5 ng/mL murine IL-3, at a concentration of 1×10^6^ cells/mL. All cytokines were produced as COS cell supernatants and were calibrated against recombinant standards. For the over-expression of Hes1 and c-Myc, LIN^-^ cells were overlaid on irradiated (1500 cGy) virus producing GP+E-86 cells contenaining the MSCV-GFP or –Hes1 or c-Myc in the presence of 0.8 µg/mL of polybrene (Sigma Aldrich) for 48h. For the down-regulation of Hes1, LIN^-^ cells were infected using lentiviral vectors containing either non-targeting shCTL or shHes1 (Sigma, TRCN0000028854; St. Louis, Missouri, United States) for 48 h. Following infection, cells were selected for 2 d with puromycin (1.5 µg/ml) and transplanted into irradiated CD45.1 hosts.

### Transcriptome sequencing of T-ALL patient samples

11 T-ALL samples were collected by the Quebec Leukemia Cell Bank with informed consent. The project was approved by the Research Ethics Board of the Maisonneuve-Rosemont Hospital and Université de Montréal. These samples include the complete array of phenotypic T-ALL, ranging from ETP (1 sample) to cortical T (3 samples), as previously published [105]. Transcriptome libraries were generated from 4 µg total RNA. Sequence data obtained by paired-end sequencing (2×100 bp, Illumina HiSeq2000) were mapped to the mouse reference genome and analyzed as reported. RNA-seq yielded 15 Gb of mapped reads per sample, with an average of 15.2 reads per kilobase per million (RPKM). Data were log2 transformed and normalized between samples. RPKM values are taken as measures of the relative molar RNA concentration for each set of transcript. Correlation coefficients calculated for *LMO2* are shown in Fig. 6C.

Additional details for clonality analysis, co-immunoprecipitation, Luciferase assays and Notch1 sequencing are provided in S1 Protocol.

## Supporting Information

S1 Fig (**A**) Schematic diagram of thymocyte differentiation. (**B**) Gating strategy for purification of thymocyte subpopulations. (**C**) Total cell numbers recovered from the thymi and spleens of mice transplanted with either pre-leukemic thymocytes or leukemic thymocytes from *SCL* ^tg^ *LMO1* ^tg^ mice. Donor thymocytes were taken during the pre-leukemic phase (5 week-old) or at time of overt leukemia (16-20 week-old).
(PDF)

S2 Fig GSI does not affect the viability of OP9-DL1 stromal cells. OP9-DL1 stromal cells were cultured in the presence (0.5–5 µM) or not of DAPT (GSI). After 4 days, the number of viable cells recovered per culture was calculated.
(PDF)

S3 Fig The *Notch1* oncogene collaborates with SCL-LMO1 to induce pre-leukemic cell infiltration in hematopoietic organs. (**A**) Mice transplanted with *SCL* ^tg^ *LMO1* ^tg^ or *Notch1* ^tg^ *SCL* ^tg^ *LMO1* ^tg^ thymocytes were analyzed by flow cytometry for reconstitution in the spleen and BM after 3 weeks (10^5^ thymocytes per mouse). (**B**) Representative FACS profiles of donor-derived T cells (CD45.2^+^Thy1^+^) recovered in the thymus, spleen and BM of mice transplanted with *SCL* ^tg^ *LMO1* ^tg^ and *Notch1* ^tg^ *SCL* ^tg^ *LMO1* ^tg^ pre-leukemic thymocytes after 3 weeks. Note that the low levels of donor-derived T cells (<1%, panel A) in the spleen and bone marrow of mice transplanted with *SCL* ^tg^ *LMO1* ^tg^ thymocytes were mature SP8 or SP4 cells whereas the thymus was repopulated to high levels (10–80%) by donor-derived immature DN and DP cells. In contrast, the spleen, bone marrow and thymus of mice transplanted with *Notch1* ^tg^ *SCL* ^tg^ *LMO1* ^tg^ pre-leukemic thymocytes were reconstituted to high levels by the same DN, ISP8 and DP cells. (**C**) Oncogenic *Notch1* did not modify the mean stem cell activities (MAS) of *SCL* ^tg^ *LMO1* ^tg^ pre-LSCs. The MAS of *SCL* ^tg^ *LMO1* ^tg^ and *Notch1* ^tg^ *SCL* ^tg^ *LMO1* ^tg^ pre-LSCs was calculated at ∼1 CRU. Box plots illustrate the medians together with the 25 and 75 percentiles and the extreme values in each distribution. (**D**) *Tcrβ* gene rearrangement signature in pre-leukemic thymocytes from *SCL^tg^LMO1^tg^*, *Notch1^tg^SCL^tg^LMO1^tg^* mice before and after transplantation.
(PDF)

S4 Fig The Notch1 oncogene confers a competitive advantage to *SCL* ^tg^ *LMO1* ^tg^ thymocytes whereas pre-TCR signalling is dispensable. (**A**) Representative FACS profiles of thymocytes from *Cd3ε*-proficient or *Cd3ε*-deficient WT mice. (**B**) DN3 *Cd3ε* ^-/-^ *SCL* ^tg^ *LMO1* ^tg^ thymocytes exhibit an aberrant self-renewal activity. Serial transplantation of pre-leukemic *Cd3ε* ^-/-^ *SCL* ^tg^ *LMO1* ^tg^ thymocytes (5×10^6^) was into primary (I), secondary (II) and tertiary (III) recipient mice (6 to 9 mice per group) (*left panel*). The absolute numbers of donor-derived DN3 thymocytes were calculated 3 weeks after transplantation (*right panel*). (**C**) The *Notch1* oncogene confers a competitive advantage to *Cd3ε^-/-^SCL-LMO1* pre-leukemic thymocytes. Illustrated are representative FACS profiles of competition assays between *Cd3ε^-/-^Notch1* ^tg^ *SCL* ^tg^ *LMO1* ^tg^ and *Cd3ε^-/-^Gfp* ^tg^ *SCL* ^tg^ *LMO1* ^tg^ thymocytes, 3 weeks post-transplantation. Data show reconstitution (Thy1.2^+^CD45.2+) within the GFP^+^ and GFP^-^ populations, representative of each cohort of transplanted recipients. (**D**) *Notch1* expands the cellular targets of *SCL-LMO1* to DN1-4 and ISP8 but not DP cells. Pre-leukemic thymocyte subsets were purified from *Notch1^tg^SCL^tg^LMO1* ^tg^ mice as indicated and transplanted at 3×10^4^ cells per recipient mouse and engraftment in the thymus, le spleena and the BM was assessed 3 weeks later.
(PDF)

S5 Fig DN3 thymocytes express highest Notch levels and exhibit highest NOTCH1 activity. (**A**) Expression levels of the *Notch1* and *Notch3* genes in purified thymocyte subsets from *WT* and *SCL* ^tg^ *LMO1* ^tg^ mice were assessed by qRT-PCR. Data are the mean +/-SD of 3 independent experiments, after normalization to *β-Actin*. (**B**) The percentages of GFP^+^ cells in thymocyte subsets from Notch1 reporter (*TNR^tg^*) mice were compared by flow cytometry analysis.
(PDF)

S6 Fig (**A**) Lineage negative (LIN^-^) cells from *SCL* ^tg^ *LMO1* ^tg^ mice (CD45.2^+^) were transduced with either MSCV-GFP, -Hes1 and -cMyc retroviral vectors as described in Fig. 3D. Absolute number of donor-derived GFP^+^CD45.2^+^ DN1, DN4 and DP thymocytes in primary mice was calculated. (**B**) *Hes1* RNA interference decreases the expansion of *Notch1* ^tg^ *SCL* ^tg^ *LMO1* ^tg^ pre-leukemic thymocytes in transplanted hosts. Lineage negative (LIN^-^) cells from *Notch1* ^tg^ *SCL* ^tg^ *LMO1* ^tg^ mice were transduced with either sh*Hes1* lentiviral vectors or non-targeted control shRNA (shCTL) and transplanted (*upper panel*). Thymocytes were harvested and transplanted into secondary recipients. Shown are the absolute numbers of donor-derived thymocyte subsets in secondary recipient mice (n = 7, ** p<0.001, *lower panel*).
(PDF)

S7 Fig (**A**) Heatmap of the 53 up-regulated genes identified by transcriptome analysis of *Cd3ε* ^-/-^ thymocytes expressing SCL-LMO1 or not with the probability of false positive <0.01 (*left panel*). Comparison of this list with the TAL-1, LMO2 and LYL1 genome binding profiles from a compendium of ChIP-seq datasets in several hematopoietic cell lines [74] (*right panel*). (**B**) *Lyl1* gene is associated with hematopoietic and cancer stem cell signature. The comparison of the up-regulated genes by SCL-LMO1 in pre-leukemic thymocytes with published gene signatures from the GeneSig and SDB databases highlights a subset of genes that are found in hematopoietic and cancer stem cell signatures, including *Lyl1*.
(PDF)

S8 Fig (**A**) GSEA analysis of E2A-bound genes in *SCL-LMO1* thymocytes was analyzed as described in Fig. 4B. (**B**) SCLm13 interacts with E47 but not LMO1. Thymocyte extracts were immunoprecipitated with the indicated antibodies (IP), followed by western blotting with the antibodies shown on the left. Note that both E47 and LMO1 co-immunoprecipitated with SCL while only E47 co-immuprecipitated with SCLm13. (**C**) The interaction between SCL and LMO1 is required for *Lyl1* promoter activation. Results are expressed as fold activation of the *Lyl1* promoter (*Lyl1*-Luc) in NIH3T3 cells co-transfected with *SCL* or *SCLm13* together with *LMO1*, *LDB1*, *E47* and *GATA1* (complex +SCL or SCLm13) relative to the reporter vector alone. The activity of this complex depends on SCL (compare complex + versus – SCL). Data were normalized to an internal control for transfection efficiency (CMV-βgal) and represent the mean ± SD (n = 3). (**D**) E protein-dependent *Ptcra* enhancer activity is similarly inhibited by SCL and SCLm13. AD10.1 DN T cells were electroporated with *Ptcra* enhancer constructs, and the MSCV vector with or without SCL or SCLm13. Results are expressed as luciferase activity relative to the minimal TATA promoter. (**E**) Loss of one *E2a* allele significantly decreased expression levels of E2A target genes in DN thymocytes. mRNA levels of *Cdkn2a* and *Ptcra* in purified DN thymocytes from *E2a* ^+/+^, *E2a* ^+/-^ and *E2a* ^-/-^ mice were determined by qRT-PCR and normalized to *β-Actin* (Mean +/- SD, n = 3).
(PDF)

S9 Fig (**A**) Pre-leukemic DN3 thymocytes from 3-week-old donor mice of the indicated genotypes were transplanted (5×10^4^ cells per recipient mouse). Donor-derived thymocytes (CD45.2^+^Thy1^+^) were analysed by flow cytometry 6 weeks post-transplantation. (**B**) Representative immunophenotypes of engrafted thymocytes of the indicated genotypes.
(PDF)

S10 Fig *LYL1-LMO1* specifically expand the DN3 cell population after transplantation. Pre-leukemic thymocytes (1.5×10^7^ cells) from 3-week-old *LYL1* ^tg^ *LMO1* ^tg^ mice (CD45.2^+^) were transplanted into sub-lethally irradiated CD45.1^+^ recipient mice. Mice were analyzed for engraftment 6 weeks post-transplantation. The expansion folds of the indicated thymocyte subsets were calculated as the ratio of the absolute numbers of donor-derived cells of each subset recovered from the thymus of transplanted mice over the absolute numbers present in the initial inoculum.
(PDF)

S11 Fig Heat map of gene expression profiles in pediatric T-ALL patient samples [38] obtained by RT-PCR.
(PDF)

S1 Table Absence of *Notch1* activating mutations in *SCL* ^tg^ *LMO1* ^tg^ and *Notch1* ^tg^ *SCL* ^tg^ *LMO1* ^tg^ pre-leukemic thymocytes. The exons 26, 27 and 34 of the *Notch1* gene from *SCL* ^tg^ *LMO1* ^tg^ and *Notch1* ^tg^ *SCL* ^tg^ *LMO1* ^tg^ pre-leukemic thymocytes before and after transplantation were sequenced. *SCL* ^tg^ *LMO1* ^tg^ leukemic cells were used as a positive control. Provided in excel file.
(XLS)

S2 Table List of NOTCH1-bound genes responding to GSI that are upregulated during thymocyte differentiation. Gene expression data from the Immgen project were collected. Listed are genes that increase by more than 1.3-fold at each transitional stage and exhibit NOTCH1-bound peaks within 2 kb of the transcription start sites [68]. Peaks that are common in 2 murine T-ALL cell lines were retained for this analysis. Provided in excel file.
(XLS)

S3 Table List of genes differentially expressed between *Cd3ε* ^-/-^ thymocytes expressing or not the *SCL* and *LMO1* oncogenes assessed by a probability of false positive threshold (Pfp) smaller than 0.01. The comparison of this list with the TAL-1/LMO2 genome binding profiles from a compendium of ChIP-seq datasets in several hematopoietic cell lines [74], identified 9 genes (in bold) that are presumed direct SCL and LMO2 targets. Provided in excel file.
(XLS)

S4 Table Significant signature enrichment in differentially expressed genes (adjusted p values ≤0.05). Provided in excel file.
(XLS)

S5 Table Sequences of oligonucleotide primers used for TaqMan Real-time quantitative PCR, *TCRβ* gene rearrangements, chromatin immunoprecipitation and for Sanger sequencing of exons 26, 27 and 34 of the *Notch1* gene. Provided in excel file.
(XLS)

S1 Protocol Additional details for clonality analysis, co-immunoprecipitation, luciferase assays and Notch1 sequencing are provided in S1 Protocol.
(DOCX)

## References

[1] Lapidot T, Sirard C, Vormoor J, Murdoch B, Hoang T, et al (1994) A cell initiating human acute myeloid leukaemia after transplantation into SCID mice. Nature 367: 645–648. 7509044 10.1038/367645a0

[2] Valent P, Bonnet D, Wohrer S, Andreeff M, Copland M, et al (2013) Heterogeneity of neoplastic stem cells: theoretical, functional, and clinical implications. Cancer Res 73: 1037–1045. 23345162 10.1158/0008-5472.CAN-12-3678

[3] Bonnet D, Dick JE (1997) Human acute myeloid leukemia is organized as a hierarchy that originates from a primitive hematopoietic cell. Nat Med 3: 730–737. 9212098 10.1038/nm0797-730

[4] Jordan CT (2009) Cancer stem cells: controversial or just misunderstood? Cell Stem Cell 4: 203–205. 19265659 10.1016/j.stem.2009.02.003 PMC2871155

[5] Vicente-Duenas C, Romero-Camarero I, Cobaleda C, Sanchez-Garcia I (2013) Function of oncogenes in cancer development: a changing paradigm. EMBO J 32: 1502–1513. 23632857 10.1038/emboj.2013.97 PMC3671260

[6] Nguyen LV, Vanner R, Dirks P, Eaves CJ (2012) Cancer stem cells: an evolving concept. Nat Rev Cancer 12: 133–143. 22237392 10.1038/nrc3184

[7] Greaves M, Maley CC (2012) Clonal evolution in cancer. Nature 481: 306–313. 22258609 10.1038/nature10762 PMC3367003

[8] Krivtsov AV, Twomey D, Feng Z, Stubbs MC, Wang Y, et al (2006) Transformation from committed progenitor to leukaemia stem cell initiated by MLL-AF9. Nature 442: 818–822. 16862118 10.1038/nature04980

[9] McCormack MP, Young LF, Vasudevan S, de Graaf CA, Codrington R, et al (2010) The Lmo2 oncogene initiates leukemia in mice by inducing thymocyte self-renewal. Science 327: 879–883. 20093438 10.1126/science.1182378

[10] Hong D, Gupta R, Ancliff P, Atzberger A, Brown J, et al (2008) Initiating and cancer-propagating cells in TEL-AML1-associated childhood leukemia. Science 319: 336–339. 18202291 10.1126/science.1150648

[11] Bhandoola A, Sambandam A (2006) From stem cell to T cell: one route or many? Nat Rev Immunol 6: 117–126. 16491136 10.1038/nri1778

[12] Martins VC, Ruggiero E, Schlenner SM, Madan V, Schmidt M, et al (2012) Thymus-autonomous T cell development in the absence of progenitor import. J Exp Med 209: 1409–1417. 22778389 10.1084/jem.20120846 PMC3420332

[13] Zhang JA, Mortazavi A, Williams BA, Wold BJ, Rothenberg EV (2012) Dynamic transformations of genome-wide epigenetic marking and transcriptional control establish T cell identity. Cell 149: 467–482. 22500808 10.1016/j.cell.2012.01.056 PMC3336965

[14] Ciofani M, Schmitt TM, Ciofani A, Michie AM, Cuburu N, et al (2004) Obligatory role for cooperative signaling by pre-TCR and Notch during thymocyte differentiation. J Immunol 172: 5230–5239. 15100261 10.4049/jimmunol.172.9.5230

[15] Weng AP, Ferrando AA, Lee W, Morris JPt, Silverman LB, et al (2004) Activating mutations of NOTCH1 in human T cell acute lymphoblastic leukemia. Science 306: 269–271. 15472075 10.1126/science.1102160

[16] O'Neil J, Calvo J, McKenna K, Krishnamoorthy V, Aster JC, et al (2006) Activating Notch1 mutations in mouse models of T-ALL. Blood 107: 781–785. 16166587 10.1182/blood-2005-06-2553 PMC1895623

[17] Tremblay M, Tremblay CS, Herblot S, Aplan PD, Hebert J, et al (2010) Modeling T-cell acute lymphoblastic leukemia induced by the SCL and LMO1 oncogenes. Genes Dev 24: 1093–1105. 20516195 10.1101/gad.1897910 PMC2878648

[18] Bigas A, Espinosa L (2012) Hematopoietic stem cells: to be or Notch to be. Blood 119: 3226–3235. 22308291 10.1182/blood-2011-10-355826

[19] Koch U, Lehal R, Radtke F (2013) Stem cells living with a Notch. Development 140: 689–704. 23362343 10.1242/dev.080614

[20] Chiang MY, Shestova O, Xu L, Aster JC, Pear WS (2012) Divergent effects of supraphysiological Notch signals on leukemia stem cells and hematopoietic stem cells. Blood. 10.1182/blood-2012-03-416503 PMC3567338 23115273

[21] Armstrong F, de la Grange PB, Gerby B, Rouyez MC, Calvo J, et al (2009) NOTCH is a key regulator of human T-cell acute leukemia initiating cell activity. Blood 113: 1730–1740. 18984862 10.1182/blood-2008-02-138172

[22] Gerby B, Clappier E, Armstrong F, Deswarte C, Calvo J, et al (2011) Expression of CD34 and CD7 on human T-cell acute lymphoblastic leukemia discriminates functionally heterogeneous cell populations. Leukemia 25: 1249–1258. 21566655 10.1038/leu.2011.93

[23] Tatarek J, Cullion K, Ashworth T, Gerstein R, Aster JC, et al (2011) Notch1 inhibition targets the leukemia-initiating cells in a Tal1/Lmo2 mouse model of T-ALL. Blood 118: 1579–1590. 21670468 10.1182/blood-2010-08-300343 PMC3156046

[24] Wendorff AA, Koch U, Wunderlich FT, Wirth S, Dubey C, et al (2010) Hes1 is a critical but context-dependent mediator of canonical Notch signaling in lymphocyte development and transformation. Immunity 33: 671–684. 21093323 10.1016/j.immuni.2010.11.014

[25] Dudley DD, Wang HC, Sun XH (2009) Hes1 potentiates T cell lymphomagenesis by up-regulating a subset of notch target genes. PLoS One 4: e6678. 19688092 10.1371/journal.pone.0006678 PMC2722736

[26] D'Altri T, Gonzalez J, Aifantis I, Espinosa L, Bigas A (2011) Hes1 expression and CYLD repression are essential events downstream of Notch1 in T-cell leukemia. Cell Cycle 10: 1031–1036. 21389783 10.4161/cc.10.7.15067 PMC3974883

[27] Weng AP, Millholland JM, Yashiro-Ohtani Y, Arcangeli ML, Lau A, et al (2006) c-Myc is an important direct target of Notch1 in T-cell acute lymphoblastic leukemia/lymphoma. Genes Dev 20: 2096–2109. 16847353 10.1101/gad.1450406 PMC1536060

[28] Steininger A, Mobs M, Ullmann R, Kochert K, Kreher S, et al (2011) Genomic loss of the putative tumor suppressor gene E2A in human lymphoma. J Exp Med 208: 1585–1593. 21788410 10.1084/jem.20101785 PMC3149217

[29] Palomero T, Lim WK, Odom DT, Sulis ML, Real PJ, et al (2006) NOTCH1 directly regulates c-MYC and activates a feed-forward-loop transcriptional network promoting leukemic cell growth. Proc Natl Acad Sci U S A 103: 18261–18266. 17114293 10.1073/pnas.0606108103 PMC1838740

[30] Chiang MY, Xu L, Shestova O, Histen G, L'Heureux S, et al (2008) Leukemia-associated NOTCH1 alleles are weak tumor initiators but accelerate K-ras-initiated leukemia. J Clin Invest 118: 3181–3194. 18677410 10.1172/JCI35090 PMC2491459

[31] Swiers G, Patient R, Loose M (2006) Genetic regulatory networks programming hematopoietic stem cells and erythroid lineage specification. Dev Biol 294: 525–540. 16626682 10.1016/j.ydbio.2006.02.051

[32] Reynaud D, Ravet E, Titeux M, Mazurier F, Renia L, et al (2005) SCL/TAL1 expression level regulates human hematopoietic stem cell self-renewal and engraftment. Blood 106: 2318–2328. 15961517 10.1182/blood-2005-02-0557

[33] Lacombe J, Herblot S, Rojas-Sutterlin S, Haman A, Barakat S, et al (2010) Scl regulates the quiescence and the long-term competence of hematopoietic stem cells. Blood 115: 792–803. 19850742 10.1182/blood-2009-01-201384

[34] Souroullas GP, Salmon JM, Sablitzky F, Curtis DJ, Goodell MA (2009) Adult hematopoietic stem and progenitor cells require either Lyl1 or Scl for survival. Cell Stem Cell 4: 180–186. 19200805 10.1016/j.stem.2009.01.001 PMC2672304

[35] Porcher C, Swat W, Rockwell K, Fujiwara Y, Alt FW, et al (1996) The T cell leukemia oncoprotein SCL/tal-1 is essential for development of all hematopoietic lineages. Cell 86: 47–57. 8689686 10.1016/s0092-8674(00)80076-8

[36] Schlaeger TM, Schuh A, Flitter S, Fisher A, Mikkola H, et al (2004) Decoding hematopoietic specificity in the helix-loop-helix domain of the transcription factor SCL/Tal-1. Mol Cell Biol 24: 7491–7502. 15314159 10.1128/MCB.24.17.7491-7502.2004 PMC506978

[37] Lecuyer E, Lariviere S, Sincennes MC, Haman A, Lahlil R, et al (2007) Protein stability and transcription factor complex assembly determined by the SCL-LMO2 interaction. J Biol Chem 282: 33649–33658. 17878155 10.1074/jbc.M703939200

[38] Ferrando AA, Neuberg DS, Staunton J, Loh ML, Huard C, et al (2002) Gene expression signatures define novel oncogenic pathways in T cell acute lymphoblastic leukemia. Cancer Cell 1: 75–87. 12086890 10.1016/s1535-6108(02)00018-1

[39] McGuire EA, Rintoul CE, Sclar GM, Korsmeyer SJ (1992) Thymic overexpression of Ttg-1 in transgenic mice results in T-cell acute lymphoblastic leukemia/lymphoma. Mol Cell Biol 12: 4186–4196. 1508213 10.1128/mcb.12.9.4186-4196.1992 PMC360323

[40] Aplan PD, Jones CA, Chervinsky DS, Zhao X, Ellsworth M, et al (1997) An scl gene product lacking the transactivation domain induces bony abnormalities and cooperates with LMO1 to generate T-cell malignancies in transgenic mice. EMBO J 16: 2408–2419. 9171354 10.1093/emboj/16.9.2408 PMC1169841

[41] Larson RC, Lavenir I, Larson TA, Baer R, Warren AJ, et al (1996) Protein dimerization between Lmo2 (Rbtn2) and Tal1 alters thymocyte development and potentiates T cell tumorigenesis in transgenic mice. Embo J 15: 1021–1027. 8605871 PMC449997

[42] Hsu HL, Wadman I, Tsan JT, Baer R (1994) Positive and negative transcriptional control by the TAL1 helix-loop-helix protein. Proc Natl Acad Sci U S A 91: 5947–5951. 8016094 10.1073/pnas.91.13.5947 PMC44114

[43] Park ST, Sun XH (1998) The Tal1 oncoprotein inhibits E47-mediated transcription. Mechanism of inhibition. J Biol Chem 273: 7030–7037. 9507011 10.1074/jbc.273.12.7030

[44] Chervinsky DS, Zhao XF, Lam DH, Ellsworth M, Gross KW, et al (1999) Disordered T-cell development and T-cell malignancies in SCL LMO1 double-transgenic mice: parallels with E2A-deficient mice. Mol Cell Biol 19: 5025–5035. 10373552 10.1128/mcb.19.7.5025 PMC84335

[45] Yan W, Young AZ, Soares VC, Kelley R, Benezra R, et al (1997) High incidence of T-cell tumors in E2A-null mice and E2A/Id1 double-knockout mice. Mol Cell Biol 17: 7317–7327. 9372963 10.1128/mcb.17.12.7317 PMC232588

[46] Herblot S, Steff AM, Hugo P, Aplan PD, Hoang T (2000) SCL and LMO1 alter thymocyte differentiation: inhibition of E2A-HEB function and pre-T alpha chain expression. Nat Immunol 1: 138–144. 11248806 10.1038/77819

[47] O'Neil J, Shank J, Cusson N, Murre C, Kelliher M (2004) TAL1/SCL induces leukemia by inhibiting the transcriptional activity of E47/HEB. Cancer Cell 5: 587–596. 15193261 10.1016/j.ccr.2004.05.023

[48] Murre C (2005) Helix-loop-helix proteins and lymphocyte development. Nat Immunol 6: 1079–1086. 16239924 10.1038/ni1260

[49] Kee BL (2009) E and ID proteins branch out. Nat Rev Immunol 9: 175–184. 19240756 10.1038/nri2507

[50] Herblot S, Aplan PD, Hoang T (2002) Gradient of E2A activity in B-cell development. Mol Cell Biol 22: 886–900. 11784864 10.1128/MCB.22.3.886-900.2002 PMC133542

[51] Goardon N, Schuh A, Hajar I, Ma X, Jouault H, et al (2002) Ectopic expression of TAL-1 protein in Ly-6E.1-htal-1 transgenic mice induces defects in B- and T-lymphoid differentiation. Blood 100: 491–500. 12091340 10.1182/blood.v100.2.491

[52] Robb L, Rasko JE, Bath ML, Strasser A, Begley CG (1995) scl, a gene frequently activated in human T cell leukaemia, does not induce lymphomas in transgenic mice. Oncogene 10: 205–209. 7824274

[53] Kelliher MA, Seldin DC, Leder P (1996) Tal-1 induces T cell acute lymphoblastic leukemia accelerated by casein kinase IIalpha. Embo J 15: 5160–5166. 8895560 PMC452259

[54] Aifantis I, Raetz E, Buonamici S (2008) Molecular pathogenesis of T-cell leukaemia and lymphoma. Nat Rev Immunol 8: 380–390. 18421304 10.1038/nri2304

[55] Kim D, Peng XC, Sun XH (1999) Massive apoptosis of thymocytes in T-cell-deficient Id1 transgenic mice. Mol Cell Biol 19: 8240–8253. 10567549 10.1128/mcb.19.12.8240 PMC84908

[56] Van Vlierberghe P, Ferrando A (2012) The molecular basis of T cell acute lymphoblastic leukemia. J Clin Invest 122: 3398–3406. 23023710 10.1172/JCI61269 PMC3461904

[57] Ono Y, Fukuhara N, Yoshie O (1998) TAL1 and LIM-only proteins synergistically induce retinaldehyde dehydrogenase 2 expression in T-cell acute lymphoblastic leukemia by acting as cofactors for GATA3. Mol Cell Biol 18: 6939–6950. 9819382 10.1128/mcb.18.12.6939 PMC109277

[58] Lecuyer E, Herblot S, Saint-Denis M, Martin R, Begley CG, et al (2002) The SCL complex regulates c-kit expression in hematopoietic cells through functional interaction with Sp1. Blood 100: 2430–2440. 12239153 10.1182/blood-2002-02-0568

[59] Kusy S, Gerby B, Goardon N, Gault N, Ferri F, et al (2010) NKX3.1 is a direct TAL1 target gene that mediates proliferation of TAL1-expressing human T cell acute lymphoblastic leukemia. J Exp Med 207: 2141–2156. 20855495 10.1084/jem.20100745 PMC2947082

[60] Sanda T, Lawton LN, Barrasa MI, Fan ZP, Kohlhammer H, et al (2012) Core transcriptional regulatory circuit controlled by the TAL1 complex in human T cell acute lymphoblastic leukemia. Cancer Cell 22: 209–221. 22897851 10.1016/j.ccr.2012.06.007 PMC3422504

[61] McCormack MP, Shields BJ, Jackson JT, Nasa C, Shi W, et al.. (2013) Requirement for Lyl1 in a model of Lmo2-driven early T-cell precursor ALL. Blood. 10.1182/blood-2012-09-458570 23926305

[62] McMurray HR, Sampson ER, Compitello G, Kinsey C, Newman L, et al (2008) Synergistic response to oncogenic mutations defines gene class critical to cancer phenotype. Nature 453: 1112–1116. 18500333 10.1038/nature06973 PMC2613942

[63] Ashton JM, Balys M, Neering SJ, Hassane DC, Cowley G, et al (2012) Gene sets identified with oncogene cooperativity analysis regulate in vivo growth and survival of leukemia stem cells. Cell Stem Cell 11: 359–372. 22863534 10.1016/j.stem.2012.05.024 PMC4023631

[64] Cancer Genome Atlas Research N (2013) Genomic and epigenomic landscapes of adult de novo acute myeloid leukemia. N Engl J Med 368: 2059–2074. 23634996 10.1056/NEJMoa1301689 PMC3767041

[65] Kalender Atak Z, Gianfelici V, Hulselmans G, De Keersmaecker K, Devasia AG, et al (2013) Comprehensive analysis of transcriptome variation uncovers known and novel driver events in T-cell acute lymphoblastic leukemia. PLoS Genet 9: e1003997. 24367274 10.1371/journal.pgen.1003997 PMC3868543

[66] Schmitt TM, Zuniga-Pflucker JC (2002) Induction of T cell development from hematopoietic progenitor cells by delta-like-1 in vitro. Immunity 17: 749–756. 12479821 10.1016/s1074-7613(02)00474-0

[67] Tan JB, Visan I, Yuan JS, Guidos CJ (2005) Requirement for Notch1 signals at sequential early stages of intrathymic T cell development. Nat Immunol 6: 671–679. 15951812 10.1038/ni1217

[68] Wang H, Zou J, Zhao B, Johannsen E, Ashworth T, et al (2011) Genome-wide analysis reveals conserved and divergent features of Notch1/RBPJ binding in human and murine T-lymphoblastic leukemia cells. Proc Natl Acad Sci U S A 108: 14908–14913. 21737748 10.1073/pnas.1109023108 PMC3169118

[69] Aster JC, Pear WS, Blacklow SC (2008) Notch signaling in leukemia. Annu Rev Pathol 3: 587–613. 18039126 10.1146/annurev.pathmechdis.3.121806.154300 PMC5934586

[70] Deneault E, Cellot S, Faubert A, Laverdure JP, Frechette M, et al (2009) A functional screen to identify novel effectors of hematopoietic stem cell activity. Cell 137: 369–379. 19379700 10.1016/j.cell.2009.03.026 PMC5770201

[71] Rossi L, Lin KK, Boles NC, Yang L, King KY, et al (2012) Less is more: unveiling the functional core of hematopoietic stem cells through knockout mice. Cell Stem Cell 11: 302–317. 22958929 10.1016/j.stem.2012.08.006 PMC3461270

[72] Sharma VM, Calvo JA, Draheim KM, Cunningham LA, Hermance N, et al (2006) Notch1 contributes to mouse T-cell leukemia by directly inducing the expression of c-myc. Mol Cell Biol 26: 8022–8031. 16954387 10.1128/MCB.01091-06 PMC1636748

[73] Kunisato A, Chiba S, Nakagami-Yamaguchi E, Kumano K, Saito T, et al (2003) HES-1 preserves purified hematopoietic stem cells ex vivo and accumulates side population cells in vivo. Blood 101: 1777–1783. 12406868 10.1182/blood-2002-07-2051

[74] Hannah R, Joshi A, Wilson NK, Kinston S, Gottgens B (2011) A compendium of genome-wide hematopoietic transcription factor maps supports the identification of gene regulatory control mechanisms. Exp Hematol 39: 531–541. 21338655 10.1016/j.exphem.2011.02.009

[75] Wilson NK, Foster SD, Wang X, Knezevic K, Schutte J, et al (2010) Combinatorial transcriptional control in blood stem/progenitor cells: genome-wide analysis of ten major transcriptional regulators. Cell Stem Cell 7: 532–544. 20887958 10.1016/j.stem.2010.07.016

[76] Lecuyer E, Hoang T (2004) SCL: from the origin of hematopoiesis to stem cells and leukemia. Exp Hematol 32: 11–24. 14725896 10.1016/j.exphem.2003.10.010

[77] Ikawa T, Kawamoto H, Goldrath AW, Murre C (2006) E proteins and Notch signaling cooperate to promote T cell lineage specification and commitment. J Exp Med 203: 1329–1342. 16682500 10.1084/jem.20060268 PMC2121213

[78] Miyazaki K, Miyazaki M, Murre C (2014) The establishment of B versus T cell identity. Trends Immunol 35: 205–210. 24679436 10.1016/j.it.2014.02.009 PMC4030559

[79] Bain G, Quong MW, Soloff RS, Hedrick SM, Murre C (1999) Thymocyte maturation is regulated by the activity of the helix-loop-helix protein, E47. J Exp Med 190: 1605–1616. 10587351 10.1084/jem.190.11.1605 PMC2195738

[80] Zhuang Y, Cheng P, Weintraub H (1996) B-lymphocyte development is regulated by the combined dosage of three basic helix-loop-helix genes, E2A, E2-2, and HEB. Mol Cell Biol 16: 2898–2905. 8649400 10.1128/mcb.16.6.2898 PMC231283

[81] Deleuze V, El-Hajj R, Chalhoub E, Dohet C, Pinet V, et al (2012) Angiopoietin-2 is a direct transcriptional target of TAL1, LYL1 and LMO2 in endothelial cells. PLoS One 7: e40484. 22792348 10.1371/journal.pone.0040484 PMC3391236

[82] Zohren F, Souroullas GP, Luo M, Gerdemann U, Imperato MR, et al (2012) The transcription factor Lyl-1 regulates lymphoid specification and the maintenance of early T lineage progenitors. Nat Immunol 13: 761–769. 22772404 10.1038/ni.2365 PMC3411897

[83] Homminga I, Pieters R, Langerak AW, de Rooi JJ, Stubbs A, et al (2011) Integrated transcript and genome analyses reveal NKX2-1 and MEF2C as potential oncogenes in T cell acute lymphoblastic leukemia. Cancer Cell 19: 484–497. 21481790 10.1016/j.ccr.2011.02.008

[84] Smith S, Tripathi R, Goodings C, Cleveland S, Mathias E, et al (2014) LIM Domain Only-2 (LMO2) Induces T-Cell Leukemia by Two Distinct Pathways. PLoS One 9: e85883. 24465765 10.1371/journal.pone.0085883 PMC3897537

[85] Homminga I, Vuerhard MJ, Langerak AW, Buijs-Gladdines J, Pieters R, et al (2012) Characterization of a pediatric T-cell acute lymphoblastic leukemia patient with simultaneous LYL1 and LMO2 rearrangements. Haematologica 97: 258–261. 22058201 10.3324/haematol.2011.051722 PMC3269487

[86] Giambra V, Jenkins CR, Wang H, Lam SH, Shevchuk OO, et al (2012) NOTCH1 promotes T cell leukemia-initiating activity by RUNX-mediated regulation of PKC-theta and reactive oxygen species. Nat Med 18: 1693–1698. 23086478 10.1038/nm.2960 PMC3738873

[87] King B, Trimarchi T, Reavie L, Xu L, Mullenders J, et al (2013) The ubiquitin ligase FBXW7 modulates leukemia-initiating cell activity by regulating MYC stability. Cell 153: 1552–1566. 23791182 10.1016/j.cell.2013.05.041 PMC4146439

[88] Maillard I, Koch U, Dumortier A, Shestova O, Xu L, et al (2008) Canonical notch signaling is dispensable for the maintenance of adult hematopoietic stem cells. Cell Stem Cell 2: 356–366. 18397755 10.1016/j.stem.2008.02.011 PMC3717373

[89] Yuan JS, Kousis PC, Suliman S, Visan I, Guidos CJ (2010) Functions of notch signaling in the immune system: consensus and controversies. Annu Rev Immunol 28: 343–365. 20192807 10.1146/annurev.immunol.021908.132719

[90] Phelan JD, Saba I, Zeng H, Kosan C, Messer MS, et al (2013) Growth factor independent-1 maintains Notch1-dependent transcriptional programming of lymphoid precursors. PLoS Genet 9: e1003713. 24068942 10.1371/journal.pgen.1003713 PMC3772063

[91] Tzoneva G, Ferrando AA (2012) Recent advances on NOTCH signaling in T-ALL. Curr Top Microbiol Immunol 360: 163–182. 22673746 10.1007/82_2012_232

[92] De Obaldia ME, Bell JJ, Wang X, Harly C, Yashiro-Ohtani Y, et al (2013) T cell development requires constraint of the myeloid regulator C/EBP-alpha by the Notch target and transcriptional repressor Hes1. Nat Immunol 14: 1277–1284. 24185616 10.1038/ni.2760 PMC4038953

[93] Laurenti E, Varnum-Finney B, Wilson A, Ferrero I, Blanco-Bose WE, et al (2008) Hematopoietic stem cell function and survival depend on c-Myc and N-Myc activity. Cell Stem Cell 3: 611–624. 19041778 10.1016/j.stem.2008.09.005 PMC2635113

[94] Baena E, Ortiz M, Martinez AC, de Alboran IM (2007) c-Myc is essential for hematopoietic stem cell differentiation and regulates Lin(-)Sca-1(+)c-Kit(-) cell generation through p21. Exp Hematol 35: 1333–1343. 17637497 10.1016/j.exphem.2007.05.015

[95] Wilson A, Murphy MJ, Oskarsson T, Kaloulis K, Bettess MD, et al (2004) c-Myc controls the balance between hematopoietic stem cell self-renewal and differentiation. Genes Dev 18: 2747–2763. 15545632 10.1101/gad.313104 PMC528895

[96] Reavie L, Della Gatta G, Crusio K, Aranda-Orgilles B, Buckley SM, et al (2010) Regulation of hematopoietic stem cell differentiation by a single ubiquitin ligase-substrate complex. Nat Immunol 11: 207–215. 20081848 10.1038/ni.1839 PMC2825759

[97] O'Neil J, Grim J, Strack P, Rao S, Tibbitts D, et al (2007) FBW7 mutations in leukemic cells mediate NOTCH pathway activation and resistance to gamma-secretase inhibitors. J Exp Med 204: 1813–1824. 17646409 10.1084/jem.20070876 PMC2118656

[98] Nakagawa M, Takizawa N, Narita M, Ichisaka T, Yamanaka S (2010) Promotion of direct reprogramming by transformation-deficient Myc. Proc Natl Acad Sci U S A 107: 14152–14157. 20660764 10.1073/pnas.1009374107 PMC2922531

[99] Roderick JE, Tesell J, Shultz LD, Brehm MA, Greiner DL, et al (2014) c-Myc inhibition prevents leukemia initiation in mice and impairs the growth of relapsed and induction failure pediatric T-ALL cells. Blood 123: 1040–1050. 24394663 10.1182/blood-2013-08-522698 PMC3924926

[100] Loosveld M, Castellano R, Gon S, Goubard A, Crouzet T, et al (2014) Therapeutic targeting of c-Myc in T-cell acute lymphoblastic leukemia, T-ALL. Oncotarget 5: 3168–3172. 24930440 10.18632/oncotarget.1873 PMC4102800

[101] Smith LJ, Curtis JE, Messner HA, Senn JS, Furthmayr H, et al (1983) Lineage infidelity in acute leukemia. Blood 61: 1138–1145. 6404327

[102] Ischenko I, Zhi J, Moll UM, Nemajerova A, Petrenko O (2013) Direct reprogramming by oncogenic Ras and Myc. Proc Natl Acad Sci U S A 110: 3937–3942. 23431158 10.1073/pnas.1219592110 PMC3593888

[103] Chiu PP, Jiang H, Dick JE (2010) Leukemia-initiating cells in human T-lymphoblastic leukemia exhibit glucocorticoid resistance. Blood 116: 5268–5279. 20810926 10.1182/blood-2010-06-292300

[104] Soulier J, Clappier E, Cayuela JM, Regnault A, Garcia-Peydro M, et al (2005) HOXA genes are included in genetic and biologic networks defining human acute T-cell leukemia (T-ALL). Blood 106: 274–286. 15774621 10.1182/blood-2004-10-3900

[105] Simon C, Chagraoui J, Krosl J, Gendron P, Wilhelm B, et al (2012) A key role for EZH2 and associated genes in mouse and human adult T-cell acute leukemia. Genes Dev 26: 651–656. 22431509 10.1101/gad.186411.111 PMC3323876

[106] Nagel S, Venturini L, Meyer C, Kaufmann M, Scherr M, et al (2010) Multiple mechanisms induce ectopic expression of LYL1 in subsets of T-ALL cell lines. Leuk Res 34: 521–528. 19608273 10.1016/j.leukres.2009.06.020

[107] Zhuang Y, Kim CG, Bartelmez S, Cheng P, Groudine M, et al (1992) Helix-loop-helix transcription factors E12 and E47 are not essential for skeletal or cardiac myogenesis, erythropoiesis, chondrogenesis, or neurogenesis. Proc Natl Acad Sci U S A 89: 12132–12136. 1465450 10.1073/pnas.89.24.12132 PMC50712

[108] Malissen M, Gillet A, Ardouin L, Bouvier G, Trucy J, et al (1995) Altered T cell development in mice with a targeted mutation of the CD3-epsilon gene. EMBO J 14: 4641–4653. 7588594 10.1002/j.1460-2075.1995.tb00146.x PMC394561

[109] Zhumabekov T, Corbella P, Tolaini M, Kioussis D (1995) Improved version of a human CD2 minigene based vector for T cell-specific expression in transgenic mice. J Immunol Methods 185: 133–140. 7665895 10.1016/0022-1759(95)00124-s

[110] Greaves DR, Wilson FD, Lang G, Kioussis D (1989) Human CD2 3'-flanking sequences confer high-level, T cell-specific, position-independent gene expression in transgenic mice. Cell 56: 979–986. 2564317 10.1016/0092-8674(89)90631-4

[111] Harrison DE, Jordan CT, Zhong RK, Astle CM (1993) Primitive hemopoietic stem cells: direct assay of most productive populations by competitive repopulation with simple binomial, correlation and covariance calculations. Exp Hematol 21: 206–219. 8425559

[112] Ema H, Nakauchi H (2000) Expansion of hematopoietic stem cells in the developing liver of a mouse embryo. Blood 95: 2284–2288. 10733497

[113] Tanigawa T, Elwood N, Metcalf D, Cary D, DeLuca E, et al (1993) The SCL gene product is regulated by and differentially regulates cytokine responses during myeloid leukemic cell differentiation. Proc Natl Acad Sci U S A 90: 7864–7868. 8356096 10.1073/pnas.90.16.7864 PMC47243

[114] Trickett A, Kwan YL (2003) T cell stimulation and expansion using anti-CD3/CD28 beads. J Immunol Methods 275: 251–255. 12667688 10.1016/s0022-1759(03)00010-3

[115] Gautier L, Cope L, Bolstad BM, Irizarry RA (2004) affy—analysis of Affymetrix GeneChip data at the probe level. Bioinformatics 20: 307–315. 14960456 10.1093/bioinformatics/btg405

[116] Breitling R, Armengaud P, Amtmann A, Herzyk P (2004) Rank products: a simple, yet powerful, new method to detect differentially regulated genes in replicated microarray experiments. FEBS Lett 573: 83–92. 15327980 10.1016/j.febslet.2004.07.055

[117] Salmon-Divon M, Dvinge H, Tammoja K, Bertone P (2010) PeakAnalyzer: genome-wide annotation of chromatin binding and modification loci. BMC Bioinformatics 11: 415. 20691053 10.1186/1471-2105-11-415 PMC2923140

[118] Tremblay M, Herblot S, Lecuyer E, Hoang T (2003) Regulation of pT alpha gene expression by a dosage of E2A, HEB, and SCL. J Biol Chem 278: 12680–12687. 12566462 10.1074/jbc.M209870200

[119] Lacombe J, Krosl G, Tremblay M, Gerby B, Martin R, et al (2013) Genetic interaction between Kit and Scl. Blood 122: 1150–1161. 23836559 10.1182/blood-2011-01-331819 PMC3952531

[120] Lachmann A, Xu H, Krishnan J, Berger SI, Mazloom AR, et al (2010) ChEA: transcription factor regulation inferred from integrating genome-wide ChIP-X experiments. Bioinformatics 26: 2438–2444. 20709693 10.1093/bioinformatics/btq466 PMC2944209

