# Supplementary figures and images for: SCL, LMO1 and Notch1 Reprogram Thymocytes into Self-Renewing Cells
Source: PLoS Genet. 2014 Dec 18;10(12):e1004768. doi: 10.1371/journal.pgen.1004768 (PMC4270438; doi:10.1371/journal.pgen.1004768)

Figure S1

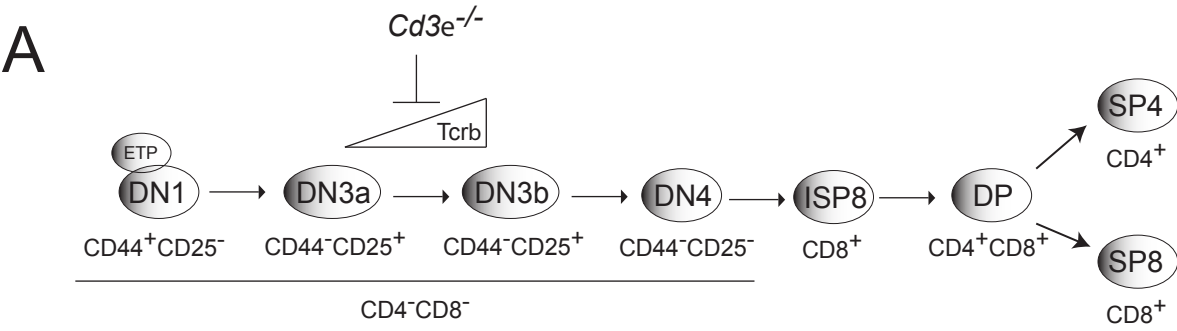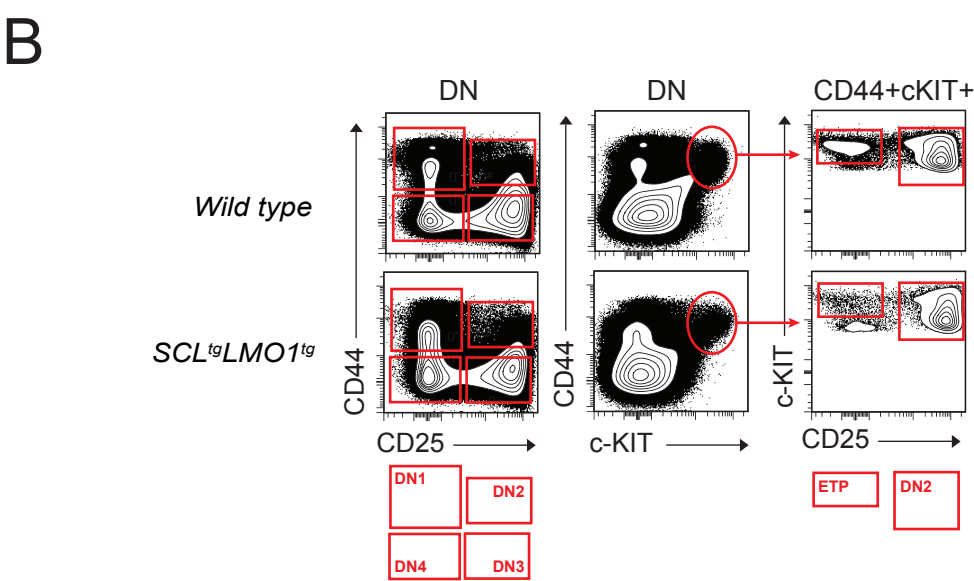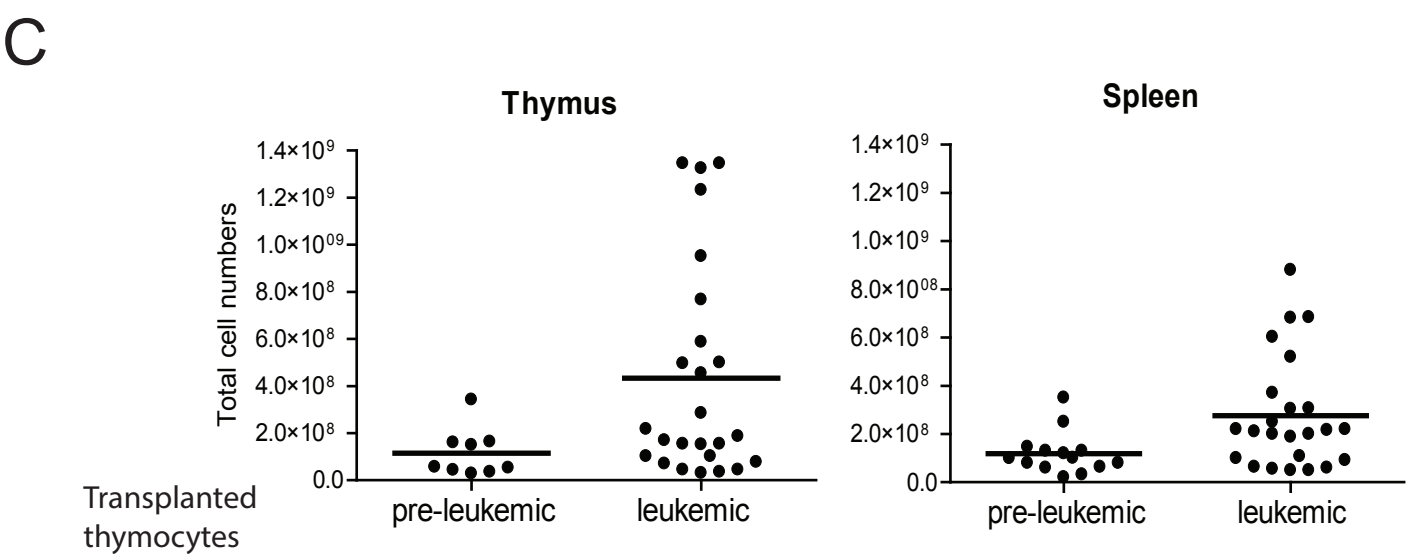

Supplement: S1 Fig — (A) Schematic diagram of thymocyte differentiation. (B) Gating strategy for purification of thymocyte subpopulations. (C) Total cell numbers recovered from the thymi and spleens of mice transplanted with either pre-leukemic thymocytes or leukemic thymocytes from SCL tg LMO1 tg mice. Donor thymocytes were taken during the pre-leukemic phase (5 week-old) or at time of overt leukemia (16-20 week-old). (PDF) [file pgen.1004768.s001.pdf]

Figure S2

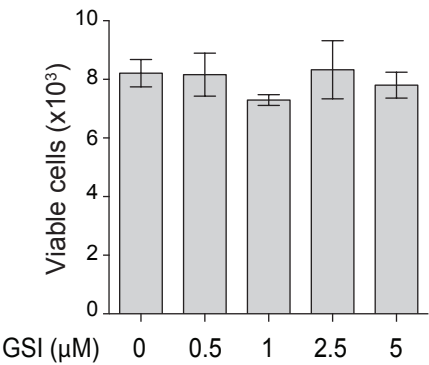

Supplement: S2 Fig — GSI does not affect the viability of OP9-DL1 stromal cells. OP9-DL1 stromal cells were cultured in the presence (0.5–5 µM) or not of DAPT (GSI). After 4 days, the number of viable cells recovered per culture was calculated. (PDF) [file pgen.1004768.s002.pdf]

# Figure S3

## A

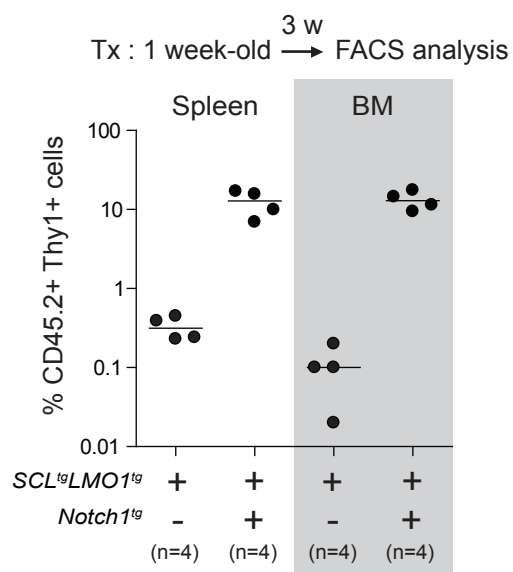

## B

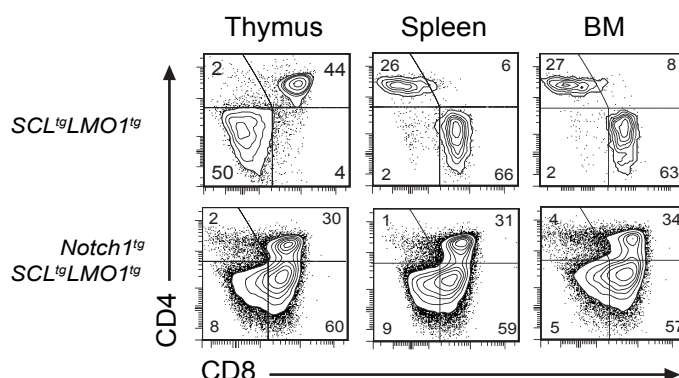

## C

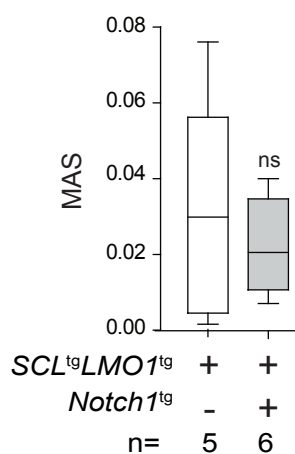

## D

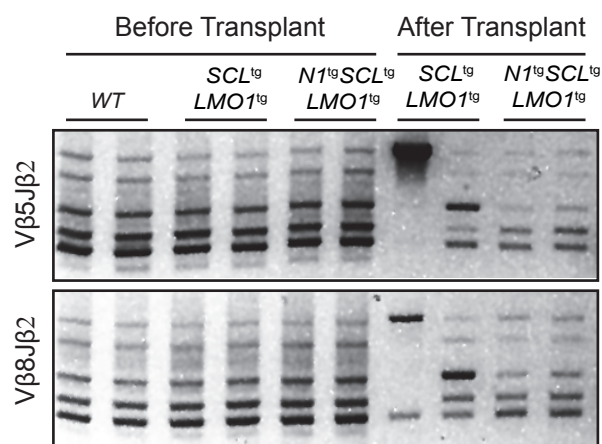

Supplement: S3 Fig — The Notch1 oncogene collaborates with SCL-LMO1 to induce pre-leukemic cell infiltration in hematopoietic organs. (A) Mice transplanted with SCL tg LMO1 tg or Notch1 tg SCL tg LMO1 tg thymocytes were analyzed by flow cytometry for reconstitution in the spleen and BM after 3 weeks (105 thymocytes per mouse). (B) Representative FACS profiles of donor-derived T cells (CD45.2+Thy1+) recovered in the thymus, spleen and BM of mice transplanted with SCL tg LMO1 tg and Notch1 tg SCL tg LMO1 tg pre-leukemic thymocytes after 3 weeks. Note that the low levels of donor-derived T cells (<1%, panel A) in the spleen and bone marrow of mice transplanted with SCL tg LMO1 tg thymocytes were mature SP8 or SP4 cells whereas the thymus was repopulated to high levels (10–80%) by donor-derived immature DN and DP cells. In contrast, the spleen, bone marrow and thymus of mice transplanted with Notch1 tg SCL tg LMO1 tg pre-leukemic thymocytes were reconstituted to high levels by the same DN, ISP8 and DP cells. (C) Oncogenic Notch1 did not modify the mean stem cell activities (MAS) of SCL tg LMO1 tg pre-LSCs. The MAS of SCL tg LMO1 tg and Notch1 tg SCL tg LMO1 tg pre-LSCs was calculated at ∼1 CRU. Box plots illustrate the medians together with the 25 and 75 percentiles and the extreme values in each distribution. (D) Tcrβ gene rearrangement signature in pre-leukemic thymocytes from SCLtgLMO1tg, Notch1tgSCLtgLMO1tg mice before and after transplantation. (PDF) [file pgen.1004768.s003.pdf]

# Figure S4

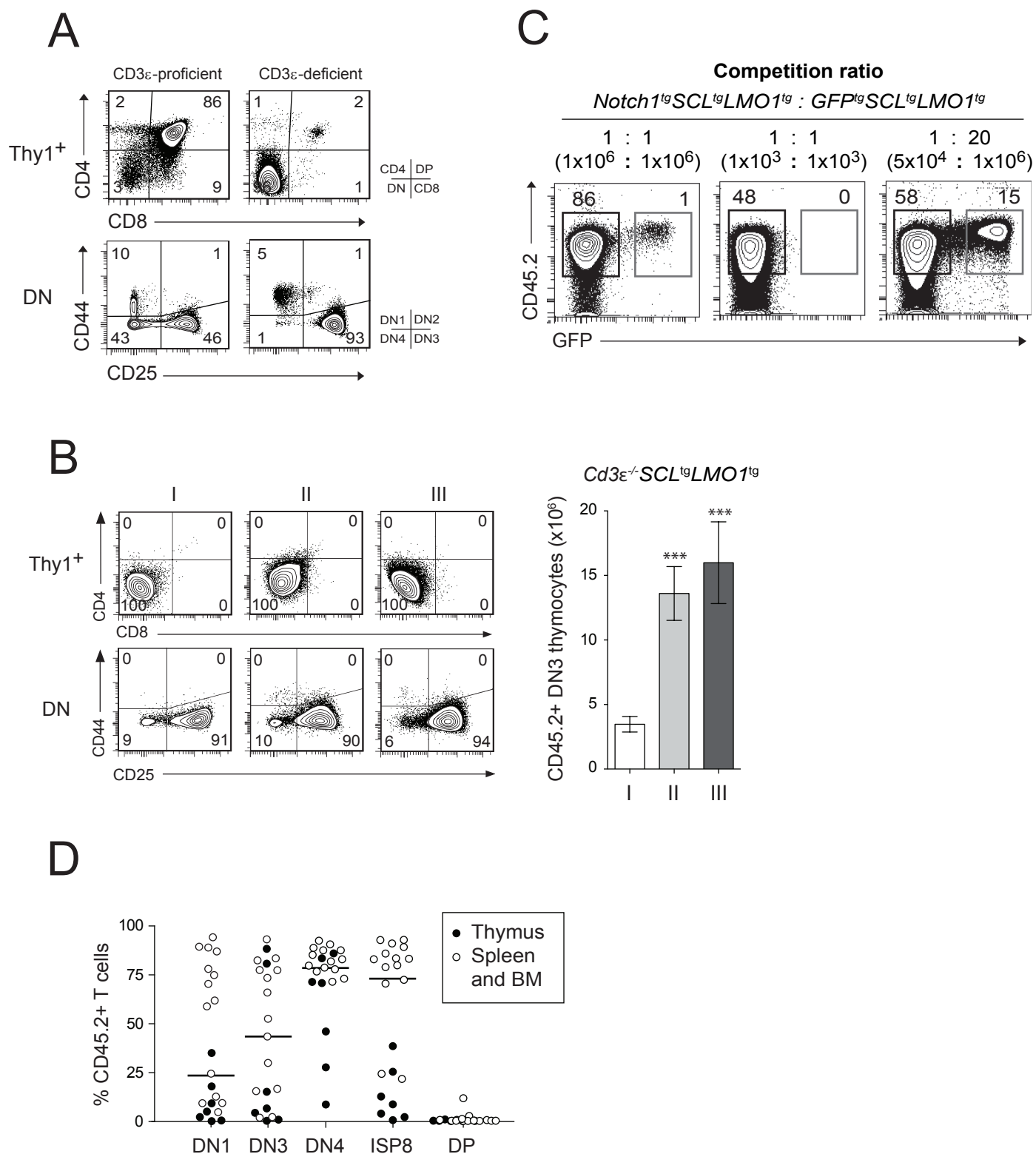

Supplement: S4 Fig — The Notch1 oncogene confers a competitive advantage to SCL tg LMO1 tg thymocytes whereas pre-TCR signalling is dispensable. (A) Representative FACS profiles of thymocytes from Cd3ε-proficient or Cd3ε-deficient WT mice. (B) DN3 Cd3ε -/- SCL tg LMO1 tg thymocytes exhibit an aberrant self-renewal activity. Serial transplantation of pre-leukemic Cd3ε -/- SCL tg LMO1 tg thymocytes (5×10^6^) was into primary (I), secondary (II) and tertiary (III) recipient mice (6 to 9 mice per group) (left panel). The absolute numbers of donor-derived DN3 thymocytes were calculated 3 weeks after transplantation (right panel). (C) The Notch1 oncogene confers a competitive advantage to Cd3ε-/-SCL-LMO1 pre-leukemic thymocytes. Illustrated are representative FACS profiles of competition assays between Cd3ε-/-Notch1 tg SCL tg LMO1 tg and Cd3ε-/-Gfp tg SCL tg LMO1 tg thymocytes, 3 weeks post-transplantation. Data show reconstitution (Thy1.2+CD45.2+) within the GFP+ and GFP- populations, representative of each cohort of transplanted recipients. (D) Notch1 expands the cellular targets of SCL-LMO1 to DN1-4 and ISP8 but not DP cells. Pre-leukemic thymocyte subsets were purified from Notch1tgSCLtgLMO1 tg mice as indicated and transplanted at 3×10^4^ cells per recipient mouse and engraftment in the thymus, le spleena and the BM was assessed 3 weeks later. (PDF) [file pgen.1004768.s004.pdf]

Figure S5

A

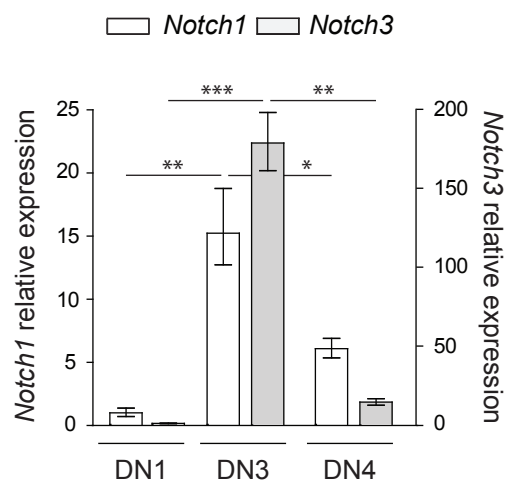

B

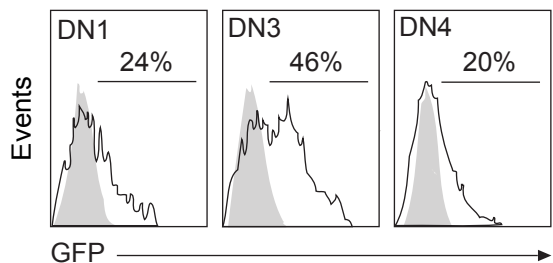

Supplement: S5 Fig — DN3 thymocytes express highest Notch levels and exhibit highest NOTCH1 activity. (A) Expression levels of the Notch1 and Notch3 genes in purified thymocyte subsets from WT and SCL tg LMO1 tg mice were assessed by qRT-PCR. Data are the mean +/-SD of 3 independent experiments, after normalization to β-Actin. (B) The percentages of GFP+ cells in thymocyte subsets from Notch1 reporter (TNRtg) mice were compared by flow cytometry analysis. (PDF) [file pgen.1004768.s005.pdf]

Figure S6

A

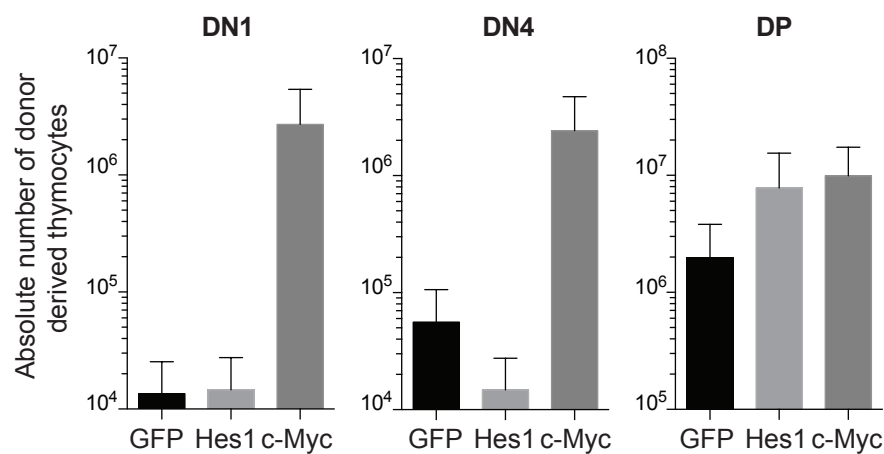

B

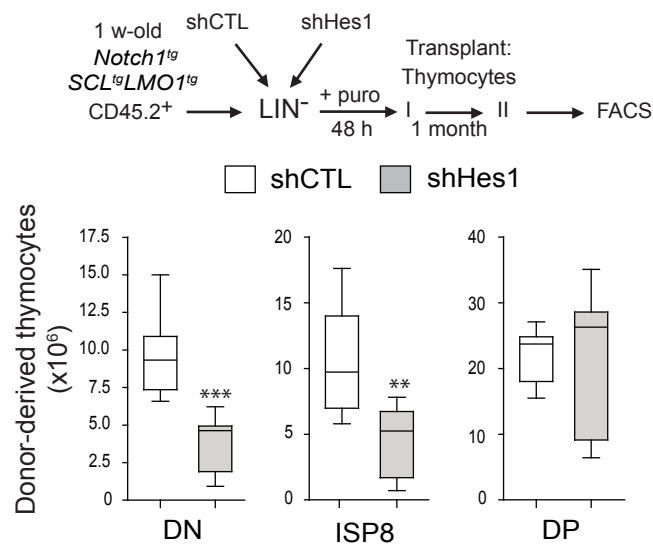

Supplement: S6 Fig — (A) Lineage negative (LIN-) cells from SCL tg LMO1 tg mice (CD45.2+) were transduced with either MSCV-GFP, -Hes1 and -cMyc retroviral vectors as described in Fig. 3D. Absolute number of donor-derived GFP+CD45.2+ DN1, DN4 and DP thymocytes in primary mice was calculated. (B) Hes1 RNA interference decreases the expansion of Notch1 tg SCL tg LMO1 tg pre-leukemic thymocytes in transplanted hosts. Lineage negative (LIN-) cells from Notch1 tg SCL tg LMO1 tg mice were transduced with either shHes1 lentiviral vectors or non-targeted control shRNA (shCTL) and transplanted (upper panel). Thymocytes were harvested and transplanted into secondary recipients. Shown are the absolute numbers of donor-derived thymocyte subsets in secondary recipient mice (n = 7, ** p<0.001, lower panel). (PDF) [file pgen.1004768.s006.pdf]

Figure S7

A

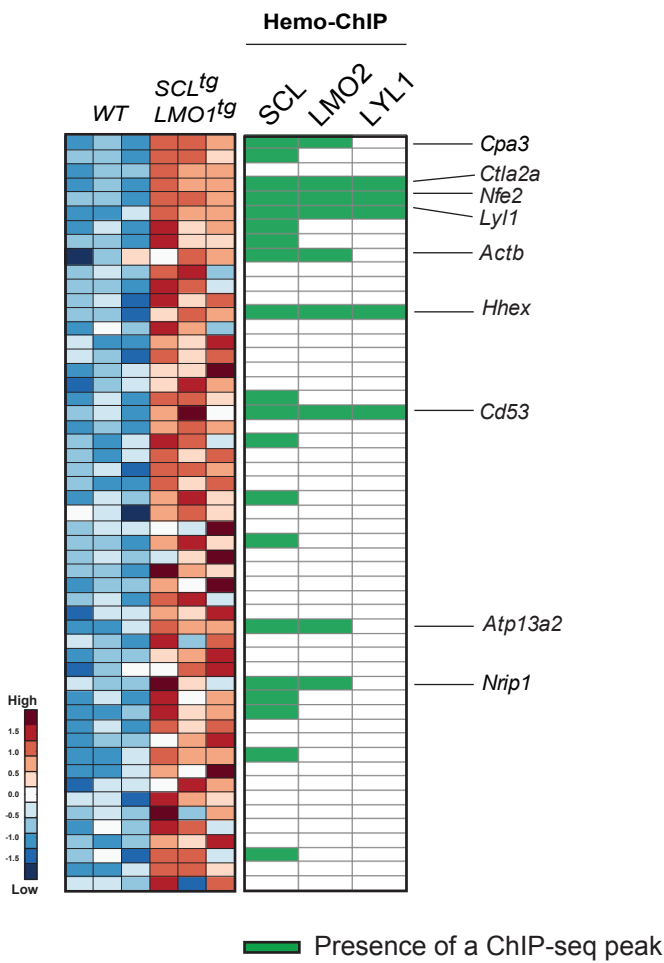

B

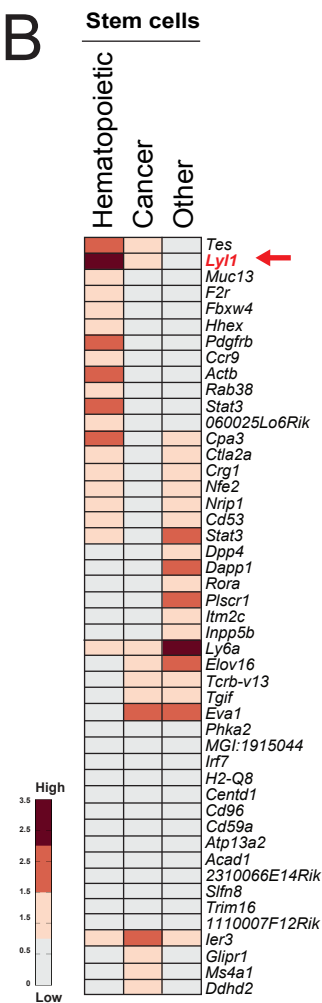

Supplement: S7 Fig — (A) Heatmap of the 53 up-regulated genes identified by transcriptome analysis of Cd3ε -/- thymocytes expressing SCL-LMO1 or not with the probability of false positive <0.01 (left panel). Comparison of this list with the TAL-1, LMO2 and LYL1 genome binding profiles from a compendium of ChIP-seq datasets in several hematopoietic cell lines [74] (right panel). (B) Lyl1 gene is associated with hematopoietic and cancer stem cell signature. The comparison of the up-regulated genes by SCL-LMO1 in pre-leukemic thymocytes with published gene signatures from the GeneSig and SDB databases highlights a subset of genes that are found in hematopoietic and cancer stem cell signatures, including Lyl1. (PDF) [file pgen.1004768.s007.pdf]

Figure S8

A

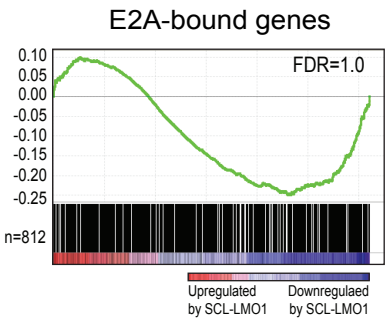

B

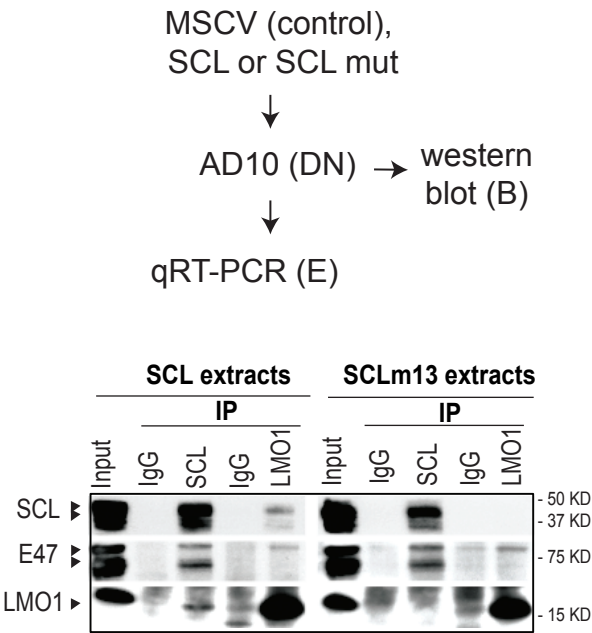

C

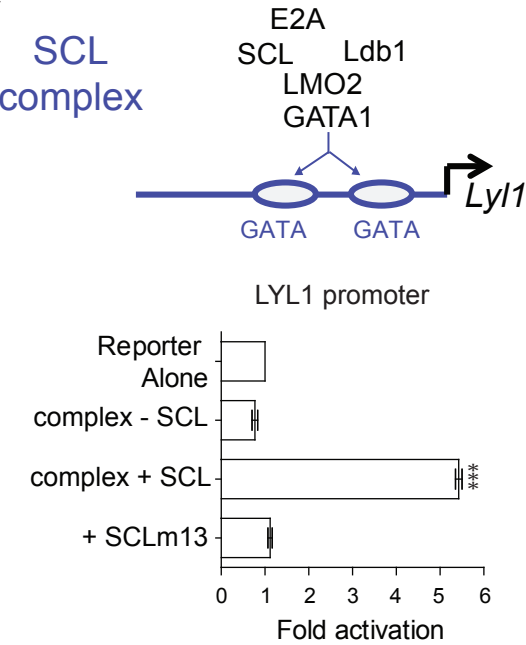

D

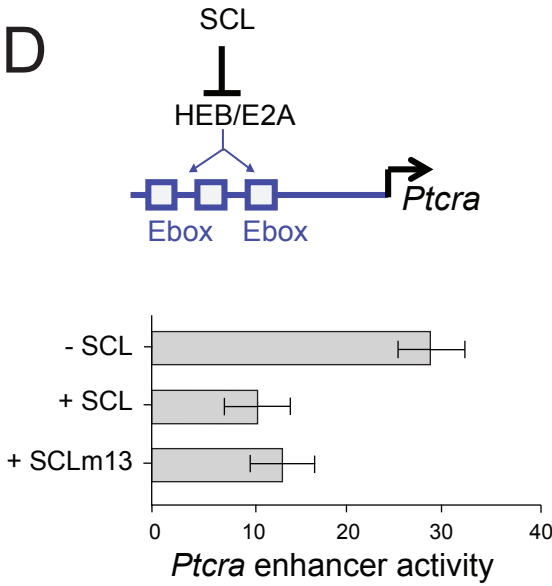

E

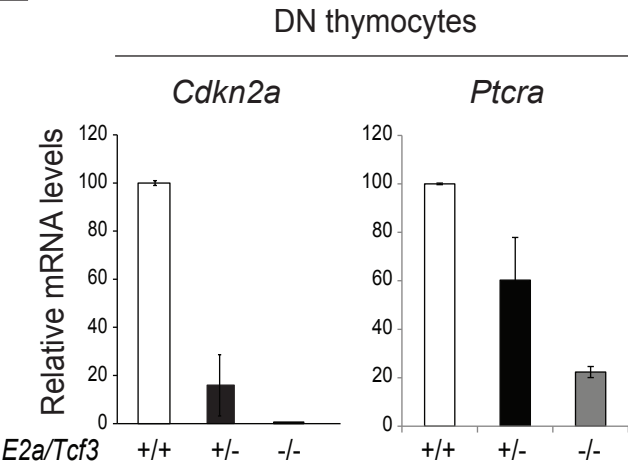

Supplement: S8 Fig — (A) GSEA analysis of E2A-bound genes in SCL-LMO1 thymocytes was analyzed as described in Fig. 4B. (B) SCLm13 interacts with E47 but not LMO1. Thymocyte extracts were immunoprecipitated with the indicated antibodies (IP), followed by western blotting with the antibodies shown on the left. Note that both E47 and LMO1 co-immunoprecipitated with SCL while only E47 co-immuprecipitated with SCLm13. (C) The interaction between SCL and LMO1 is required for Lyl1 promoter activation. Results are expressed as fold activation of the Lyl1 promoter (Lyl1-Luc) in NIH3T3 cells co-transfected with SCL or SCLm13 together with LMO1, LDB1, E47 and GATA1 (complex +SCL or SCLm13) relative to the reporter vector alone. The activity of this complex depends on SCL (compare complex + versus – SCL). Data were normalized to an internal control for transfection efficiency (CMV-βgal) and represent the mean ± SD (n = 3). (D) E protein-dependent Ptcra enhancer activity is similarly inhibited by SCL and SCLm13. AD10.1 DN T cells were electroporated with Ptcra enhancer constructs, and the MSCV vector with or without SCL or SCLm13. Results are expressed as luciferase activity relative to the minimal TATA promoter. (E) Loss of one E2a allele significantly decreased expression levels of E2A target genes in DN thymocytes. mRNA levels of Cdkn2a and Ptcra in purified DN thymocytes from E2a +/+, E2a +/- and E2a -/- mice were determined by qRT-PCR and normalized to β-Actin (Mean +/- SD, n = 3). (PDF) [file pgen.1004768.s008.pdf]

Figure S9

A

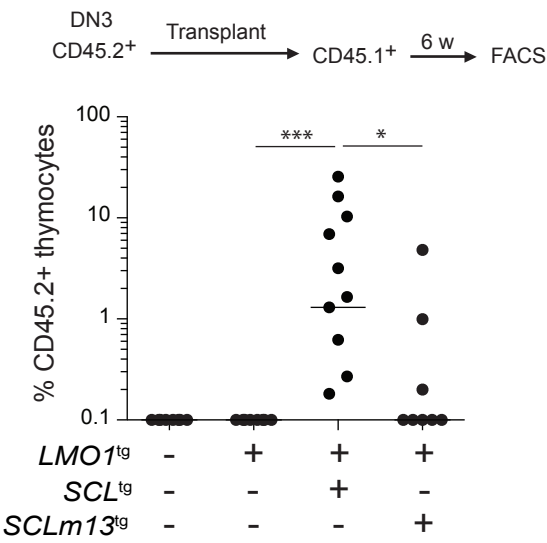

B

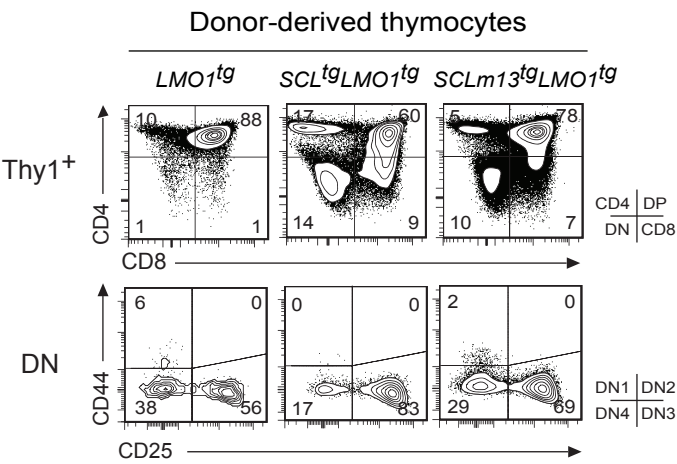

Supplement: S9 Fig — (A) Pre-leukemic DN3 thymocytes from 3-week-old donor mice of the indicated genotypes were transplanted (5×10^4^ cells per recipient mouse). Donor-derived thymocytes (CD45.2+Thy1+) were analysed by flow cytometry 6 weeks post-transplantation. (B) Representative immunophenotypes of engrafted thymocytes of the indicated genotypes. (PDF) [file pgen.1004768.s009.pdf]

Figure S10

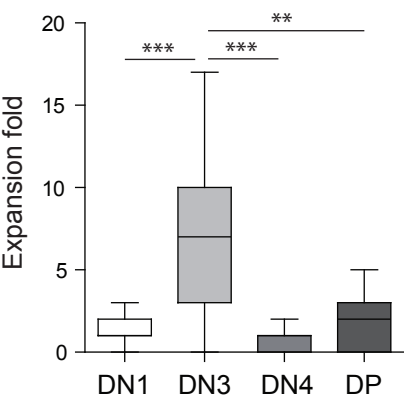

Supplement: S10 Fig — LYL1-LMO1 specifically expand the DN3 cell population after transplantation. Pre-leukemic thymocytes (1.5×10^7^ cells) from 3-week-old LYL1 tg LMO1 tg mice (CD45.2+) were transplanted into sub-lethally irradiated CD45.1+ recipient mice. Mice were analyzed for engraftment 6 weeks post-transplantation. The expansion folds of the indicated thymocyte subsets were calculated as the ratio of the absolute numbers of donor-derived cells of each subset recovered from the thymus of transplanted mice over the absolute numbers present in the initial inoculum. (PDF) [file pgen.1004768.s010.pdf]

Figure S11

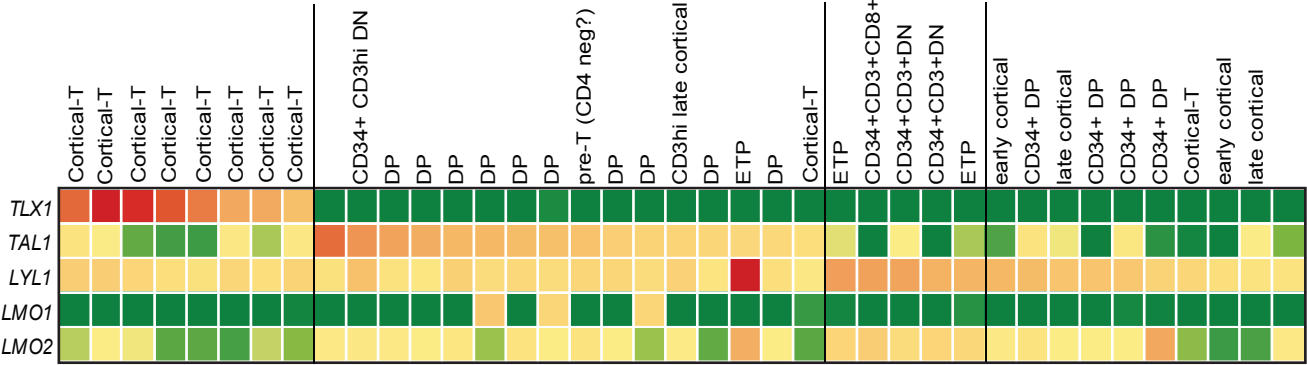

Supplement: S11 Fig — Heat map of gene expression profiles in pediatric T-ALL patient samples [38] obtained by RT-PCR. (PDF) [file pgen.1004768.s011.pdf]
