# Supplementary material for: SCL, LMO1 and Notch1 Reprogram Thymocytes into Self-Renewing Cells
Source: PLoS Genet. 2014 Dec 18;10(12):e1004768. doi: 10.1371/journal.pgen.1004768 (PMC4270438; doi:10.1371/journal.pgen.1004768)
Supplement: S1 Protocol — Additional details for clonality analysis, co-immunoprecipitation, luciferase assays and Notch1 sequencing are provided in S1 Protocol. (DOCX) [file pgen.1004768.s017.docx]

**Supplemental Experimental procedures**

Clonality analysis

Genomic DNA from pre-leukemic cells was extracted using the DNAzol Reagent (10503-027, Invitrogen, Burlington, Ontario, Canada), followed by ethanol precipitation. *Tcrβ* gene rearrangements (Vβ5Jβ2 and Vβ8Jβ2) were determined by PCR amplification [[40](#_ENREF_40)]. Primer sequences are listed in Table S5.

Co-Immunoprecipitation assays

293 cell nuclear extracts were incubated overnight at 4 °C with 3 μg of antibody in 1 mL of IP buffer (20 mM Tris-HCl (pH 8.0), 137 mM NaCl, 1% Nonidet P-40, 10% glycerol, 1 mM EDTA). Protein complexes were precipitated by adding appropriately conjugated Pansorbin cells (Calbiochem, Etobicoke, Ontario, Canada) for 30–120 min at 4 °C, washed 3 times with 1 ml of IP buffer, and subjected to SDS-PAGE. After transfer on PVDF membranes, proteins were visualized by immunoblotting (IB) using ECL plus (GE Healthcare; Burnaby, British Columbia, Canada). The mouse anti-E47 (YAE) was from Santa Cruz Biotechnology Inc (Santa Cruz, California, United States). The rabbit anti-LMO1 (A300-314-A) was from Bethyl Laboratories (Cedarlane Laboratories, Burlington, Ontario, Canada). The BTL-73 mouse anti-human SCL was provided by D. Mathieu-Mahul.

Luciferase assays

The *Gypa-84* [[124](#_ENREF_124)] or *Lyl1* [[125](#_ENREF_125)] promoters (1500 ng) were transfected in NIH 3T3 cells with expression vectors for E47 (150 ng), LMO2 (750 ng), Ldb-1 (750 ng) and GATA-1 (150 ng), and the indicated amounts of SCL vectors (150 ng) as previously described [[58](#_ENREF_58)]. Calcium phosphate was used to transfect NIH 3T3 cells 24 h after plating at 30,000 cells per milliliter. The amount of reporter was kept at 1.5 μg per well, and 100 ng cytomegalovirus-βgal (CMV-βgal) was added as an internal control. Total DNA was kept constant at 4.5 μg per well with pGem4. Luciferase and βgal activities were assayed after 48 h. For all transfections, results are shown as the mean ± SD of replicate determinations of 2 or more independent experiments. The AD10.1 immature T cell line (CD3^-^CD4^-^CD8^-^) was electroporated with the following plasmids: *Ptcra* enhancer element [[121](#_ENREF_121)], and the MSCV empty vector, MSCV-SCL expressing vector or SCLm13 mutant. Results are expressed as luciferase activity relative to the minimal TATA promoter and represent the average ± S.D. of replicate determinations and are representative of 3 independent experiments. Luciferase reporter activities were normalized to that of an internal control (CMV-βgal).

*Notch1* sequencing

Amplification of Notch1 exons 26, 27, and 34 from genomic DNA of *SCL*^tg^*LMO1*^tg^ and *Notch1*^tg^ *SCL*^tg^*LMO1*^tg^ pre-leukemic thymocytes before and after transplantation was performed by PCR. *SCL*^tg^*LMO1*^tg^ leukemic cells were a positive control. Amplification products were sequenced in both directions. Primer sequences are shown in Table S5. Residues are numbered according to their location in Notch1 sequence (Ensembl sequence ENSMUSG00000026923).
